# Ethnomycological study on wild mushrooms in Pu’er Prefecture, Southwest Yunnan, China

**DOI:** 10.1186/s13002-022-00551-7

**Published:** 2022-08-10

**Authors:** Ran Wang, Mariana Herrera, Wenjun Xu, Peng Zhang, Jesús Pérez Moreno, Carlos Colinas, Fuqiang Yu

**Affiliations:** 1 Department of Crop and Forest Science, University of Lleida, Av. Alcalde Rovira Roure, 191, 25198 Lleida, Spain; 2 Germplasm Bank of Wild Species, Yunnan Key Laboratory for Fungal Diversity and Green Development, Kunming Institute of Botany, Chinese Academy of Sciences, 132 Lanhei Road, Kunming, 650201 Yunnan People’s Republic of China; 3 Plant Science and Conservation, Chicago Botanic Garden, 1000 Lake Cook Road, Glencoe, IL USA; 4 Department of Agriculture, Graduate School of Science and Technology, Shinshu University, 8304 Minami-minowa, Kamiina, Nagano Japan; 5 Edafología, Campus Montecillo, Colegio de Postgraduados, Km 36.5 Carr. México-Texcoco, CP 56230 Montecillo, Texcoco, Estado de México Mexico; 6 Forest Sciences Center of Catalonia (CTFC), Crta. Sant Llorenç S/N, Solsona, Spain

**Keywords:** Ethnomycology, Fungal diversity, Pu’er, South of the Tropic of Cancer

## Abstract

**Background:**

Yunnan is rich in fungal diversity and cultural diversity, but there are few researches on ethnomycology. In addition, extensive utilization of wild edible fungi (WEF), especially the ectomycorrhizal fungi, threatens the fungal diversity. Hence, this study aims to contribute to the ethnomycological knowledge in Pu’er Prefecture, Yunnan, China, including information on the fungal taxa presented in markets and natural habitats, with emphasis in ectomycorrhizal fungi (EMF).

**Methods:**

Semi-structured interviews with mushroom vendors in markets and with mushroom collectors in natural habitats were conducted. Information related to local names, habitat, fruiting time, species identification, price, cooking methods and preservation methods of wild edible mushrooms were recorded. Wild edible fungi were collected from forests, and morphological and molecular techniques were used to identify fungal species.

**Results:**

A total of 11 markets were visited during this study. The 101 species collected in the markets belonged to 22 families and 39 genera, and about 76% of them were EMF. A wealth of ethnomycological knowledge was recorded, and we found that participants in the 45–65 age group were able to judge mushroom species more accurately. Additionally, men usually had a deepest mushroom knowledge than women. A total of 283 species, varieties and undescribed species were collected from natural habitats, and about 70% of them were EMF. Mushroom species and recorded amounts showed correspondence between markets and the natural habitats on different months.

**Conclusion:**

The present study shows that Pu’er Prefecture is rich in local mycological knowledge and fungal diversity. However, it is necessary to continue the research of ethnomycological studies and to design and conduct dissemination of local knowledge in order to preserve it, since it currently remains mainly among the elderly population.

## Background

Wild edible fungal fruiting bodies, or mushrooms, known as “delicacies from the mountains,” are a natural forest resource widely acknowledged for their nutritional, medicinal, economic and cultural value [1–4]. China is one of the most important mushroom producers in the world in terms of the total volume of trade and commercialized fungal species. The Yunnan Province in southwestern China, in particular, has an important tradition of consumption and mushroom trade [5]. In China, there are about 900 species of wild edible fungi (WEF), 90% of which are present in Yunnan and utilized by local people as both a source of food and income [6]. Most of the main mushroom markets, with a large variety of species, are located in the central regions of the province because of the dense population, convenient transportation and high market demand, while countless small mushroom markets with unique fungal species are scattered throughout Yunnan in mountainous areas which are inhabited by a number of ethnic groups [7] where gathering of WEF and mushroom industry has become an important tool for poverty alleviation [8–10].

The rural population of Yunnan has a wealth of traditional knowledge related to WEF and is familiar with many species as well as their uses and ecology. The traditional mycological knowledge, generally gathered by the indigenous communities in their long interaction with nature, is an important part of human cultural heritage [11–15]. Ethnomycology is a relatively new area of research that investigates traditional knowledge, as well as cultural and environmental effects, of the association between human societies and fungi [16]. Yunnan is the province with the largest number of ethnic groups in China, each minority with their own culture, language, history and, of course, different uses for wild forest fungi. Brown [17] investigated Yi ethnomycological knowledge in four communities in Nanhua County, Yunnan Province, which showed that documenting ethnomycological knowledge highlights the importance of fungi in local ecosystems and livelihoods. Ethnomycological knowledge is a key tool for forest conservation to predict anthropic harvesting pressure zones of WEF and support the management and sustainable utilization of wild fungi [18]. For example, documenting the fungal biodiversity which has a local use would allow to design and implement strategies to cultivate the most important WEF in specific areas and at the same time to integrate this cultivation into production systems which contribute to the recycling of local agricultural wastes, providing at the same time nutritious and healthy food. Additionally, the record of the local ethnomycological knowledge would allow to increase the promotion of responsible use and to design preservation techniques for the most valuable WEF, in order to maintain this important natural resource as a livelihood opportunity in rural areas. Additionally, the documentation and preservation of traditional mycological knowledge are fundamental to avoid poisonings [19]. However, compared with local folk knowledge related to plants and animals, ethnomycological knowledge started late and remains scarce [20, 21].

The annual production of WEF in Yunnan amounts to about 80,000 t [6]. The largest market share of commercial fungi, either in terms of monetary value or of quantity, includes truffles (*Tuber indicum* Cooke & Massee, *Tuber sinoaestivum* J.P. Zhang & P.G. Liu), matsutake (*Tricholoma matsutake* (S. Ito & S. Imai) Singer), porcini (*Boletus edulis* Bull.), chanterelles (*Cantharellus cibarius* Fr.) and milk agaric (e.g., *Lactarius deliciosus* (L.) Gray, *Lactifluus volemus* (Fr.) Kuntze). Most of high-priced WEF are ectomycorrhizal fungi (EMF) which form a symbiotic relationship with trees and play an important role in the ecosystem [22, 23]. Wang and Liu [7] studied systematically the trade of fungi in Yunnan markets and showed that about 81.2% of the WEF species are EMF. Limited by cultivation techniques, mushrooms, especially of EMF, have been almost exclusively harvested from the wild [24]. Their high economic value has been driving forest-dependent communities to completely devote their resources to hunting mushrooms for immediate cash thanks to an endless market demand [25, 26]. Disorderly digging and hunting, habitat loss, and vegetation deterioration, has caused overexploitation of many species and is threatening the survival of fungal populations and the forests that support them [27–30]. A survey of mushroom markets and natural habitats in Yunnan, to a large extent, will reveal the problems of development and utilization of WEF [31, 32].

Based on this scenario, in the present research we studied the areas in Pu’er Prefecture in the southern part of Yunnan Province which has the highest diversity of both cultures and fungi. Yu et al. [33] studied the species diversity, use and threatened status of WEF in two counties of Pu’er Prefecture and found that large-scale commercial harvesting had led to the decline of mushroom production. Pu’er Prefecture has an area of 45,385 km^2^, and its population is 2.4 million. It is located in southwest Yunnan and bordered by Myanmar, Laos and Vietnam. The Tropic of Cancer runs through the middle of Pu’er. It generally belongs to subtropical monsoon climate with lower altitude, diverse topography, rich forest resources and unique ethnic groups, like Hani, Lahu, Wa or Dai.

In this study, we aimed (1) to gather ethnomycological knowledge regarding the fungal species used by ethnic groups in Pu’er; (2) to update the knowledge about fungal species sold in Pu’er markets, especially ectomycorrhizal species; (3) to document the fungal diversity inhabiting Pu’er forests through natural habitats sampling; and (4) to identify the fungal species (sold in markets and collected in the natural habitats) using taxonomical and molecular approaches.

## Methods

### Study area

**Fig. 1:**
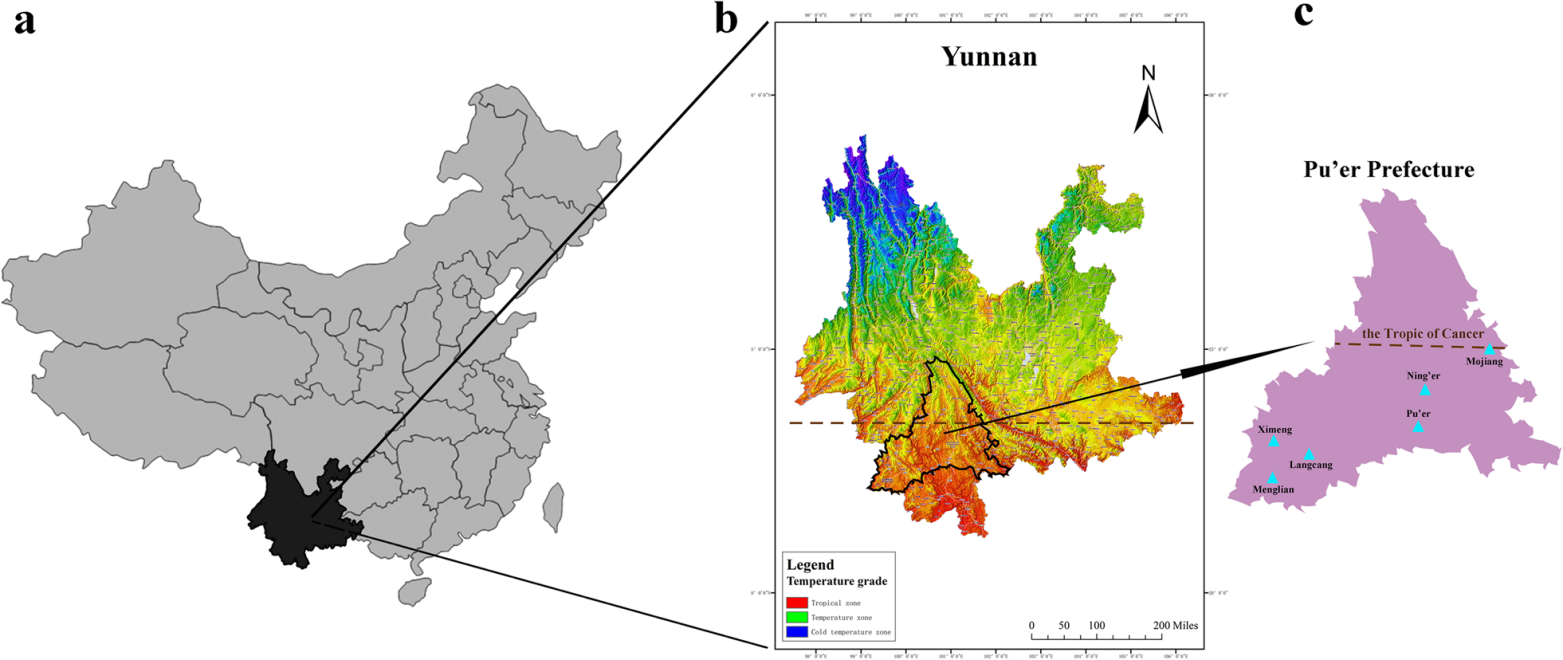
Location of study sites in six counties, Pu’er Prefecture, Yunnan Province, Southwest China. **a** China; **b** Yunnan Province; **c** Pu’er Prefecture

Pu’er Prefecture, with a total area of 45,385 square kilometers, is the largest prefecture in Yunnan Province. It is located between 22°02' N–24°50' N and 99°09' E–102°19' E, and the Tropic of Cancer runs across the middle of the prefecture. About 62.8% of Pu’er is forested where the main type of vegetation is broad-leaved forest, mixed forest (*Alnus*, *Castanopsis*, *Olea*, *Pinus*, *Quercus*) and *Pinus* forests [34]. Pu’er is one of the most culturally diverse prefectures, with 2.4 million population and fourteen ethnic groups inhabiting this area. Our investigation was carried out in five nationality autonomous counties (Lancang Lahu, Menglian Dai Lahu Wa, Mojiang Hani, Ning’er Hani Yi, Ximeng Wa) and one homonymous municipality, Pu’er (Fig. 1, Table 1), all located south of the Tropic of Cancer.

**Table 1:** Sociodemographic characteristics of the six studied localities in Pu’er Prefecture

Locality	Population	Main ethnic groups	Economy
Pu’er Municipality	416,200	Hani, Yi, Lahu	Agriculture, tea, robber, animal
Mojiang County	281,600	Hani, Yi, Dai	Agriculture, tea, walnut, tobacco, animal
Ning’er County	162,700	Hani, Yi, Dai	Agriculture, tea, fruit, animal
Lancang County	441,500	Lahu, Wa, Hani	Agriculture, tea, animal
Ximeng County	87,300	Wa, Lahu, Dai	Agriculture, tea, robber, coffee, walnut
Menglian County	144,700	Lahu, Wa, Dai	Agriculture, tea, robber, coffee

### Ethnomycological survey in markets

**Fig. 2:**
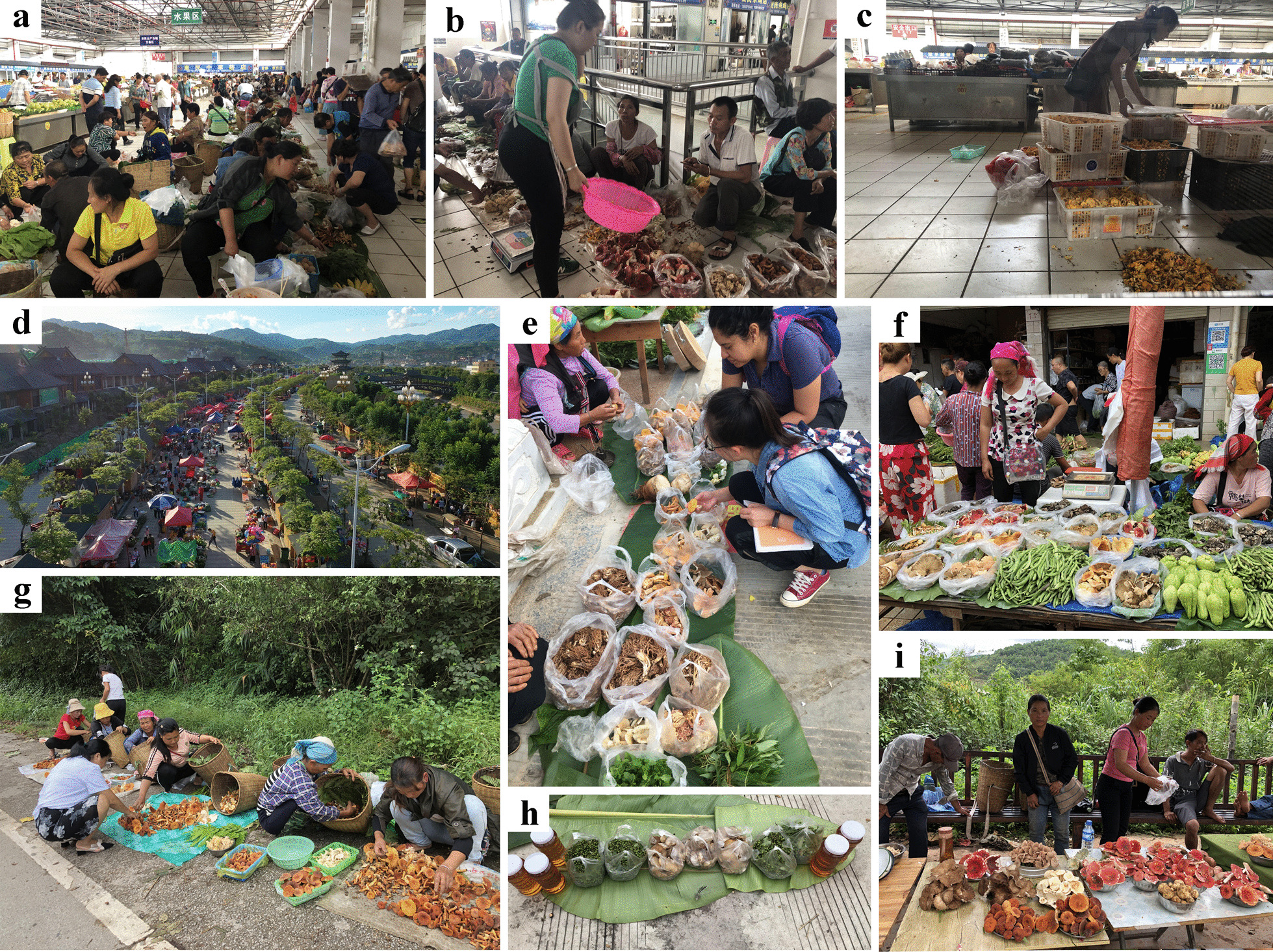
Sampled markets. Two big markets: **a**–**c** Wuyi Market, Pu’er Municipality; **d**, **e** Lancang Street Market, Lancang County; **f**–**i** Some small markets in Mojiang, Ning’er, Ximeng and Menglian counties

Semi-structured interviews were carried during the mushroom season (July to October) in three consecutive years (2019 to 2021) in established mushroom markets, mobile markets and street-stalls beside county highways or village roads (Fig. 2, Table 2). The number and the male–female ratio of vendors in markets, the knowledge, attitude and practice of human–mushroom interaction including the local names of mushrooms and their local uses (medicine, food, etc.), habitat, seasonality of species, marketability, form of mushrooms used (fresh/dried), methods of preparation for food and preservation (storage) were also recorded. For illiterate vendors, interviews were carried out mainly in Mandarin Chinese, although local languages were also used with assistance from local guides. Twenty percent of vendors in markets were randomly selected as respondents to answer the semi-structured interviews. Obtained information from these interviews was written down in sheets, which avoided distrust in the interviewed people.

**Table 2:** The timetable of selling mushrooms, minority and the average number of vendors with different gender in markets in three years

Markets’ name	Type of market^1^	Business Hours	Ethnic groups	July		August		September		October	
				Female	Male	Female	Male	Female	Male	Female	Male
Wuyi, Pu’er market	EM	2 p.m–6 p.m	Hani, Yi, Lahu	47	19	172	56	141	46	14	9
Lancang street	EM	7 a.m–12 p.m on Sunday	Lahu, Hani, Yi	44	17	93	25	183	56	55	15
Mojiang market	EM	1 p.m–4 p.m	Hani, Yi	10	2	67	37	15	8	4	1
Ning’er market	MM	2 p.m–5 p.m	Yi, Hani	17	5	49	13	51	27	26	10
Menglian market	MM	7 a.m–11 a.m every five days	Lahu, Dai, Wa	3	2	8	2	23	5	10	4
Ximeng market	MM	4 p.m–8 p.m	Wa, Lahu, Dai	2	1	2	8	41	7	10	3
No name	SS^2^	1 pm–5 p.m	×	×	×	×	×	×	×	×	×

^1^Type of markets. EM is established market, MM is mobile market, SS is street-stall

^2^Street-stalls beside county highways or village roads. We only recorded information about business time because of strong mobility

### Diversity of culturally relevant wild fungi in forests

In order to record the vegetation types associated with the fungal species sold in the markets and to investigate the presence of additional edible fungal species different than those recorded in the markets or with other uses and relevance categories, WEF were collected from forests nearby the studied markets in Pu’er prefecture. The forest areas were selected according to the information provided by some collectors previously interviewed in the markets. Forests nearby visited markets, reforested areas and a national nature reserve (Table 3) were investigated. Field work was conducted during the same season as the interviews were carried out using the random line transect method [35]. In order to gather more ethnomycological information regarding WEF, participant observation was performed in some forest areas. We joined some collectors in their daily routine of collecting WEF. While walking with them, we recorded some local names of the mushrooms, hours invested in this activity, types of collectors and habitat ecological information.

**Table 3:** Description of the sampling sites in natural habitats

Location	Altitude (m)	Locality	Forest type	Habitat
Pu’er Municipality	1450	22°49′13" N, 101°00′12"	Forests nearby markets	Pure pine forests (*Pinus kesiya*)
	1608	22°60′38''N, 101°09′65''E	The Sun River National Forest Park	Mixed forests (*Pinus*, *Quercus*, *Castanopsis*, *Olea*)
Mojiang County	1595	23°22′48.85"N, 101°41′0.69"E	Forests nearby markets	Mixed forests (*Pinus*, *Quercus*)
	1627	23°44′43.00"N, 101°12′36.1"E	Ecological forest (Kuaifa village)	Pure pine forests (*P*. *kesiya*)
Ning’er County	1437	23°0′11.35"N, 100°59′47.6"E	Forests nearby markets	Mixed forests (*Pinus*, *Quercus*)
	1537	22′59′50.84"N, 101°0′19.18"E	Ecological forest (Hualiang village)	Pure pine forests (*P*. *kesiya*)
Lancang County	1350	22°19′51''N, 100°00′34''E	Forests nearby markets	Mixed forests (*Quercus*, *Alnus*, *Pinus*)
	1490	22°35′02''N, 99°58′44''E	Forests nearby markets	Mixed forests (*Quercus*, *Pinus*)
Ximeng County	1128	22°37′14.06"N, 99°35′53.98"E	Forests nearby markets	Mixed forests (*Quercus*, *Alnus*, *Pinus*)
	1497	22°36′10.53"N, 99°35′0.02"E	Forests nearby markets	Mixed forests (*Quercus*, *Alnus*, *Pinus*)
Menglian County	1250	22°16′21.99"N, 99°16′30.06"E	Forests nearby markets	Mixed forests (*Quercus*, *Alnus*, *Pinus*)
	1380	22°16′46.11"N, 99°16′27.97"E	Forests nearby markets	Mixed forests (*Quercus*, *Alnus*, *Pinus*)

### Morphological study

Collections purchased from markets and collected from natural habitats were identified through taxonomical and molecular studies. Morpho-anatomical descriptions based on fresh samples were obtained following Largent [36]. A small sample of tissue, mostly hymenophore, was stored in silica gel and/or frozen in Eppendorf’s tubes and stored at − 20 °C to be used later for molecular analyses. Then, all the samples were dried in a hot air dehydrator at 45 °C for further analyses. All collections were deposited in the Herbarium of Cryptogams, Kunming Institute of Botany, Chinese Academy of Sciences (HKAS). Microscopic characteristics were described from fresh specimens. Dried samples were sectioned with a razor blade by hand, mounted in 5% KOH solution and then stained with Melzer’s reagent. The sections were examined under a compound light microscope (Leica DM2500).

### DNA extraction, PCR amplification and sequencing

DNA of samples was extracted using an Aidlab™ kit (Beijing). The internal transcribed spacer (ITS) region of the ribosomal DNA was amplified from DNA extracts using the ITS1F/ITS4 primer pair [37, 38]. To amplify the ribosomal large subunit (LSU), the primer combination of LROR and LR5 [39] was used. Each 25 μL PCR mixture consisted of 2.5 μL 10 × PCR buffer (Mg^2+^), 1.5 μL dNTPs (1 mM), 1 μL BSA (0.1%), 1 μL each primer (5 μM), 1 μL 25-fold diluted DNA extracts (obtained following the manufacturer’s instructions), 0.5 μL MgCl_2_ (25 mM) and 1.5 U Taq DNA polymerase (Takara, Takara Biotechnology, Dalian Co. Ltd, China). The amplifications were performed with the following cycling parameters for ITS: 94 °C for 5 min, followed by 35 cycles of 94 °C for 1 min, 50 °C for 1 min and 72 °C for 1 min, and with a final extension at 72 °C for 10 min. The amplifications were performed with the following cycling parameters for LSU: 94 °C for 3 min, followed by 35 cycles of 94 °C for 1 min, 50 °C for 1.5 min and 72 °C for 2 min, and with a final extension at 72 °C for 10 min. Three microliters of each PCR product were run on 1% (w/v) agarose gels and stained with ethidium bromide. The PCR products were purified and sequenced forward and reverse sequences at TsingKe Biological Technology, Kunming, China, using ITS1F/ITS4 and LROR/LR5 primer pairs. Sequences were edited manually using Sequencher™ 4.1.4 (Gene Codes, USA) and queried against the NCBI public database GenBank with the BLASTn algorithm for identification. Sequences generated in this study were deposited in GenBank.

## Results

### Diversity of wild mushrooms in markets and in the natural habitats

#### Update and supplement of mushroom species

A total of 623 (HKAS 106765–HKAS 122601) samples were obtained and identified. From those, 110 were collected from markets and 513 from the natural habitats. A total of 310 wild mushroom species, varieties and some undescribed species which are currently under taxonomic study along with ethnomycological catalog information such as scientific names, family names, ecology and edibility were recorded (Table 4). No significant changes were recorded in the amount or diversity of commercialized species during the sampling period. Edibility information of most of the mushrooms was gathered directly from sellers and confirmed by taxonomists, professional atlases [40–43] and specialized literature. The 310 recorded species belong to 56 families and 112 genera. Approximately 70% of the species are ectomycorrhizal. Among of them, the 101 species collected in the markets belong to 22 families and 39 genera, and about 76% of them are EMF. The 283 species collected in the natural habitats belong to 52 families and 100 genera, and about 70% are EMF.

**Table 4:** List of the mushroom species observed and acquired in the 3 years of the study at the markets and forests

Scientific name	Family name	Market	Natural habitat	ECM	Edible part	Voucher No
*Abortiporus biennis* (Bull.) Singer	Podoscyphaceae	**√**			Inedible, wood-decay fungus	HKAS-111766
*Acervus globulosus* Ekanayaka, Q. Zhao & K.D. Hyde	Pyronemataceae		**√**		Inedible, too tiny	HKAS-122632
*Agaricus heterocystis* Heinem. & Gooss.-Font	Agaricaceae		**√**		Edible	HKAS-122370
*Agaricus luteofibrillosus* M.Q. He, Linda J. Chen & R.L. Zhao	Agaricaceae		**√**		Edible	HKAS-122412
*Agaricus* sp*.*	Agaricaceae		**√**		Unknown	HKAS-122511
*Albatrellus* sp.	Albatrellaceae		**√**	√	Edible	HKAS-111880
*Amanita albidostipes* Y.Y. Cui, Q. Cai & Zhu L. Yang	Amanitaceae		**√**	√	Toxic	HKAS-124004
*Amanita angustilamella* (Höhn.) Boedijn	Amanitaceae		**√**	√	Unknown	HKAS-123967
*Amanita caojizong* Zhu L. Yang, Y.Y. Cui & Q. Cai	Amanitaceae	**√**	**√**	**√**	Edible	HKAS-124005
*Amanita* cf. *griseofarinosa*	Amanitaceae		**√**	**√**	Unknown	HKAS-122658
*Amanita citrinoannulata* Y.Y. Cui, Q. Cai & Zhu L. Yang	Amanitaceae		**√**	√	Toxic	HKAS-122410
*Amanita elata* (Massee) Corner & Bas	Amanitaceae		**√**	√	Maybe toxic	HKAS-123968
*Amanita esculenta* Hongo & I. Matsuda	Amanitaceae		**√**	√	Toxic	HKAS-122372
*Amanita eijii* Zhu L. Yang	Amanitaceae		**√**	√	Unknown	HKAS-111744
*Amanita fritillaria* (Sacc.) Sacc	Amanitaceae		**√**	√	Toxic	HKAS-111691
*Amanita griseofolia* Zhu L. Yang	Amanitaceae		**√**	√	Edible	HKAS-111779
*Amanita levistriata* D.T. Jenkins	Amanitaceae		**√**	√	Toxic	HKAS-111778
*Amanita princeps* D.T. Jenkins	Amanitaceae		**√**	√	Toxic	HKAS-122502
*Amanita pseudoporphyria* Hongo	Amanitaceae		**√**	√	Toxic	HKAS-111708
*Amanita pseudovaginata* Hongo	Amanitaceae		**√**	√	Unknown	HKAS-122692
*Amanita rubescens* Pers	Amanitaceae		**√**	√	Toxic	HKAS-122544
*Amanita rubromarginata* Har. Takah. Zhu L. Yang	Amanitaceae	**√**	**√**	√	Edible	HKAS-122664
*Amanita rubrovolvata* S. Imai	Amanitaceae		**√**	√	Toxic	HKAS-122702
*Amanita rufoferruginea* Hongo	Amanitaceae		**√**	**√**	Toxic	HKAS-111723
*Amanita sinensis* Zhu L. Yang	Amanitaceae	**√**	**√**	**√**	Edible	HKAS-122507
*Amanita spissacea* S. Imai	Amanitaceae		**√**	**√**	Toxic	HKAS-111877
*Amanita subglobosa* Zhu L. Yang	Amanitaceae		**√**	**√**	Maybe toxic	HKAS-122396
*Amanita subhemibapha* Zhu L. Yang, Y.Y. Cui & Q. Cai	Amanitaceae		**√**	**√**	Edible	HKAS-122503
*Amanita sychnopyramis* Corner & Bas	Amanitaceae		**√**	**√**	Toxic	HKAS-122650
*Amanita virgineoides* Bas	Amanitaceae		**√**	√	Maybe toxic	HKAS-111833
*Amanita yuaniana* Zhu L. Yang	Amanitaceae		**√**	**√**	Edible	HKAS-122505
*Amanita zonata* Y.Y. Cui, Qing Cai & Zhu L. Yang	Amanitaceae		**√**	**√**	Maybe toxic	HKAS-122624
*Amauroderma rugosum* (Blume & T. Nees) Torrend	Ganodermataceae	**√**	**√**		Medicinal	HKAS-111701
*Anamika angustilamellata* Zhu L. Yang & Z.W. Ge	Hymenogastraceae		**√**	**√**	Maybe toxic	HKAS-111783
*Asterophora lycoperdoides* (Bull.) Ditmar	Lyophyllaceae		**√**		Unknown	HKAS-122678
*Aureoboletus mirabilis* (Murrill) Halling	Boletaceae		**√**	**√**	Edible	HKAS-123972
*Auricularia delicata* (Mont. ex Fr.) Henn	Auriculariaceae	**√**	**√**		Edible	HKAS-111857
*Auricularia fuscosuccinea* (Mont.) Henn	Auriculariaceae	**√**			Edible	HKAS-122598
*Blastosporella zonata* T.J. Baroni & Franco-Mol	Lyophyllaceae		**√**	**√**	Unknown	HKAS-111854
*Boletellus indistinctus* G. Wu, Fang Li & Zhu L. Yang	Boletaceae	**√**	**√**	**√**	Edible	HKAS-111749
*Boletus* sp1	Boletaceae		**√**	**√**	Unknown	HKAS-111715
*Boletus* sp2	Boletaceae		**√**	**√**	Unknown	HKAS-111794
*Boletus* sp3	Boletaceae		**√**	**√**	Unknown	HKAS-122405
*Boletus aereus* Bull	Boletaceae	**√**		**√**	Edible	HKAS-124009
*Boletus auripes* Peck	Boletaceae	**√**		**√**	Edible	HKAS-111826
*Boletus bainiugan* Dentinger	Boletaceae	**√**		**√**	Edible	HKAS-111821
*Boletus monilifer* B. Feng, Y.Y. Cui, J.P. Xu & Zhu L. Yang	Boletaceae		**√**	**√**	Edible	HKAS-111704
*Boletus reticulatus* Schaeff	Boletaceae	**√**	**√**	**√**	Edible	HKAS-122381
*Boletus subvelutipes* Peck	Boletaceae	**√**		**√**	Edible	HKAS-111756
*Boletus violaceofuscus* W.F. Chiu	Boletaceae		**√**	**√**	Edible	HKAS-123966
*Bondarzewia berkeleyi* (Fr.) Bondartsev & Singer	Bondarzewiaceae		**√**		Unknown	HKAS-122722
*Butyriboletus peckii* (Frost) Kuan Zhao & Zhu L. Yang	Boletaceae	**√**		**√**	Edible, but sour or bitter	HKAS-111872
*Butyriboletus huangnianlaii* N.K. Zeng, H. Chai & Zhi Q. Liang	Boletaceae	**√**		**√**	Edible	HKAS-111755
*Caloboletus yunnanensis* Kuan Zhao & Zhu L. Yang	Boletaceae		**√**	**√**	Edible	HKAS-122727
*Cantharellus albovenosus* Buyck, Antonín & Ryoo	Hydnaceae	**√**	**√**	**√**	Edible	HKAS-123957
*Cantharellus amethysteus* (Quél.) Sacc	Hydnaceae	**√**	**√**	**√**	Edible	HKAS-111841
*Cantharellus appalachiensis* R.H. Petersen	Hydnaceae	**√**	**√**	**√**	Edible	HKAS-123956
*Cantharellus cibarius* Fr	Hydnaceae	**√**	**√**	**√**	Edible	HKAS-123958
*Cantharellus cinnabarinus* (Schwein.) Schwein	Hydnaceae	**√**	**√**	**√**	Edible	HKAS-111815
*Cantharellus* sp1	Hydnaceae		**√**	**√**	Edible	HKAS-111824
*Cantharellus* sp2	Hydnaceae		**√**	**√**	Edible	HKAS-124011
*Cantharellus tabernensis* Feib. & Cibula	Hydnaceae		**√**	**√**	Edible	HKAS-111856
*Cantharellus yunnanensis* W.F. Chiu	Hydnaceae	**√**	**√**	**√**	Edible	HKAS-123959
*Cantharellus vaginatus* S.C. Shao, X.F. Tian & P.G. Liu	Hydnaceae	**√**	**√**	**√**	Edible	HKAS-111852
*Ceriporiopsis semisupina* C.L. Zhao, B.K. Cui & Y.C. Dai	Meruliaceae		**√**		Unknown	HKAS-111855
*Cerrena zonata* (Berk.) H.S.Yuan	Cerrenaceae		**√**		Unknown	HKAS-122586
*Clarkeinda trachodes* (Berk.) Singer	Agaricaceae		**√**		Toxic	HKAS-122723
*Clavaria zollingeri* Lév	Clavariaceae		**√**		Inedible, contains lectin	HKAS-111865
*Clavulina alpina* Franchi & M. Marchetti	Hydnaceae		**√**	**√**	Edible	HKAS-122671
*Clavulina cristata* (Holmsk.) J. Schröt	Hydnaceae		**√**	**√**	Edible	HKAS-111850
*Clavulina flava* (Holmsk.) J. Schröt	Hydnaceae		**√**	**√**	Maybe edible	HKAS-122481
*Clavulina rugosa* (Bull.) J. Schröt	Hydnaceae		**√**	**√**	Edible	HKAS-111717
*Clavulina* sp.	Hydnaceae		**√**	**√**	Maybe edible	HKAS-122494
*Clavulinopsis fusiformis* (Sowerby) Corner	Clavariaceae		**√**		Edible	HKAS-122627
*Clitopilus chalybescens* T.J. Baroni & Desjardin	Entolomataceae		**√**		Unknown	HKAS-111784
*Clitopilus sinoapalus* S.P. Jian & Zhu L. Yang	Entolomataceae		**√**		Unknown	HKAS-122631
*Clitopilus* sp.	Entolomataceae		**√**		Unknown	HKAS-122655
*Collybiopsis fibrosipes* (Berk. & M.A. Curtis) R.H. Petersen	Marasmiaceae		**√**		Unknown	HKAS-122635
*Coltricia crassa* Y.C. Dai	Marasmiaceae		**√**		Inedible, dry and tough	HKAS-122441
*Coltricia weii* Y.C. Dai	Hymenochaetaceae		**√**	**√**	Inedible, dry and tough	HKAS-122593
*Cordyceps militaris* (L.) Fr	Cordycipitaceae		**√**		Medicinal	HKAS-111869
*Cordyceps nutans* Pat	Cordycipitaceae		**√**		Medicinal	HKAS-122491
*Cortinarius* aff. *torvus*	Cortinariaceae		**√**	**√**	Unknown	HKAS-122452
*Cortinarius albocyaneus* Fr	Cortinariaceae		**√**	**√**	Unknown	HKAS-111851
*Cortinarius alpinus* Boud	Cortinariaceae		**√**	**√**	Unknown	HKAS-122660
*Cortinarius boulderensis* A.H. Sm	Cortinariaceae		**√**	**√**	Unknown	HKAS-122445
*Cortinarius caesiifolius* A.H. Sm	Cortinariaceae		**√**	**√**	Unknown	HKAS-122446
*Cortinarius cotoneus* Fr	Cortinariaceae		**√**	**√**	Edible	HKAS-122455
*Cortinarius croceus* (Schaeff.) Gray	Cortinariaceae		**√**	**√**	Unknown	HKAS-122559
*Cortinarius fulvo-ochrascens* Rob. Henry	Cortinariaceae		**√**	**√**	Unknown	HKAS-122657
*Cortinarius picoides* Soop	Cortinariaceae		**√**	**√**	Edible	HKAS-111713
*Cortinarius purpurascens* Fr	Cortinariaceae		**√**	**√**	Edible	HKAS-122529
*Cortinarius* sp.	Cortinariaceae		**√**	**√**	Unknown	HKAS-111771
*Cortinarius tenuipes* (Hongo) Hongo	Cortinariaceae	**√**		**√**	Edible	HKAS-122467
*Cortinarius trivialis* J.E. Lange	Cortinariaceae		**√**	**√**	Unknown	HKAS-111789
*Cortinarius valgus* Fr	Cortinariaceae		**√**	**√**	Unknown	HKAS-111836
*Cortinarius vinaceobrunneus* Ammirati, Beug, Liimat., Niskanen & O. Ceska	Cortinariaceae		**√**	**√**	Unknown	HKAS-122626
*Craterellus aureus* Berk. & M.A. Curtis	Hydnaceae	**√**		**√**	Edible	HKAS-123973
*Craterellus cornucopioides* (L.) Pers	Hydnaceae	**√**	**√**	**√**	Edible	HKAS-111827
*Craterellus luteus* T.H. Li & X.R. Zhong	Hydnaceae		**√**	**√**	Edible	HKAS-111759
*Craterellus parvogriseus* U. Singh, K. Das & Buyck	Hydnaceae		**√**	**√**	Edible	HKAS-122486
*Craterellus* sp.	Hydnaceae	**√**	**√**	**√**	Edible	HKAS-122643
*Craterellus tubaeformis* (Fr.) Quél	Hydnaceae		**√**	**√**	Edible	HKAS-111843
*Crocinoboletus laetissimus* (Hongo) N.K. Zeng, Zhu L. Yang & G. Wu	Boletaceae	**√**	**√**	**√**	Edible	HKAS-122417
*Crocinoboletus* sp.	Boletaceae	**√**	**√**	**√**	Edible	HKAS-111764
*Cyptotrama asprata* (Berk.) Redhead & Ginns	Physalacriaceae		**√**		Unknown	HKAS-122721
*Entocybe trachyospora* (Largent) Largent, T.J. Baroni & V. Hofst	Entolomataceae		**√**		Maybe toxic	HKAS-122647
*Entoloma omiense* (Hongo) E. Horak	Entolomataceae		**√**		Toxic	HKAS-111709
*Entoloma petchii* E. Horak	Entolomataceae		**√**		Maybe toxic	HKAS-122493
*Entoloma praegracile* Xiao L. He & T.H. Li	Entolomataceae		**√**		Maybe toxic	HKAS-111787
*Entoloma subsinuatum* Murrill	Entolomataceae		**√**		Maybe toxic	HKAS-122542
*Entoloma* sp.	Entolomataceae		**√**		Unknown	HKAS-111834
*Fistulina hepatica* (Schaeff.) With	Fistulinaceae		**√**		Edible, but acidic and slightly bitter	HKAS-111775
*Fistulina* sp.	Fistulinaceae		**√**		Unknown	HKAS-111893
*Fistulina subhepatica* B.K. Cui & J. Song	Fistulinaceae		**√**		Unknown	HKAS-122466
*Fomitopsis pinicola* (Sw.) P. Karst	Fomitopsidaceae		**√**		Medicinal	HKAS-111896
*Ganoderma lingzhi* Sheng H. Wu, Y. Cao & Y.C. Dai	Polyporaceae	**√**	**√**		Medicinal	HKAS-111736
*Geastrum velutinum* Morgan	Geastraceae		**√**	**√**	Unknown	HKAS-111879
*Gerronema xanthophyllum* (Bres.) Norvell, Redhead & Ammirati	Marasmiaceae		**√**		Unknown	HKAS-122652
*Gloeophyllum sepiarium* (Wulfen) P. Karst	Gloeophyllaceae		**√**		Medicinal	HKAS-122703
*Gomphus orientalis* R.H. Petersen & M. Zang	Gomphaceae	**√**		**√**	Edible	HKAS-111823
*Gymnopilus penetrans* (Fr.) Murrill	Hymenogastraceae		**√**		Toxic	HKAS-122710
*Gymnopus dryophilus* (Bull.) Murrill	Omphalotaceae		**√**		Edible, but not worthwhile because of thin flesh and tough stem	HKAS-122640
*Gymnopus subnudus* (Ellis ex Peck) Halling	Omphalotaceae		**√**		Unknown	HKAS-122729
*Gyrodon* sp.	Paxillaceae		**√**		Unknown	HKAS-122638
*Gyroporus longicystidiatus* Nagas. & Hongo	Gyroporaceae		**√**		Edible	HKAS-122449
*Harrya chromipes* (Frost) Halling, Nuhn, Osmundson & Manfr. Binder	Boletaceae	**√**		**√**	Edible	HKAS-123979
*Hebeloma angustilamellatum* (Zhu L. Yang & Z.W. Ge) B.J. Rees	Hymenogastraceae		**√**	**√**	Unknown	HKAS-122492
*Hebeloma crustuliniforme* (Bull.) Quél	Hymenogastraceae		**√**	**√**	Toxic	HKAS-122681
*Hebeloma parvisporum* Sparre Pedersen, Læssøe, Beker & U. Eberh	Hymenogastraceae	**√**	**√**	**√**	Edible	HKAS-111767
*Heimioporus conicus* N.K. Zeng & Zhu L. Yang	Boletaceae		**√**	**√**	Toxic	HKAS-122685
*Heimioporus japonicus* (Hongo) E. Horak	Boletaceae	**√**	**√**	**√**	Toxic, but sold in market	HKAS-111748
*Heinemannomyces splendidissimus* Watling	Agaricaceae		**√**	**√**	Unknown	HKAS-111897
*Hourangia nigropunctata* (W.F. Chiu) Xue T. Zhu & Zhu L. Yang	Boletaceae		**√**	**√**	Maybe toxic	HKAS-111700
*Hydnum albidum* Peck	Hydnaceae		**√**	**√**	Edible	HKAS-111707
*Hydnum berkeleyanum* K. Das, Hembrom, A. Baghela & Vizzin	Hydnaceae	**√**	**√**	**√**	Edible	HKAS-122362
*Hydnum repandum* K. Das, Hembrom, A. Baghela & Vizzini	Hydnaceae	**√**	**√**	**√**	Edible	HKAS-111770
*Hydnum rufescens* pers	Hydnaceae	**√**	**√**	**√**	Edible	HKAS-122528
*Hydnum* sp.	Hydnaceae	**√**		**√**	Edible	HKAS-111800
*Hygrocybe cantharellus* (Schwein.) Murrill	Hygrophoraceae		**√**		Edible, but not worthwhile. Because it is too tiny	HKAS-124010
*Hygrocybe coccineocrenata* (P.D. Orton) M.M. Moser	Hygrophoraceae		**√**		Unknown	HKAS-124006
*Hygrocybe conica var. conica*	Hygrophoraceae		**√**		Maybe toxic	HKAS-111878
*Hygrocybe cuspidata* (Peck) Murrill	Hygrophoraceae	**√**	**√**		Unknown	HKAS-124008
*Hymenochaete subferruginea* Bres. & Syd	Hymenochaetaceae		**√**		Unknown	HKAS-122472
*Hymenopellis orientalis* (R.H. Petersen & Nagas.) R.H. Petersen	Physalacriaceae		**√**		Edible	HKAS-111710
*Hypomyces chlorinigenus* Rogerson & Samuels	Hypocreaceae		**√**		Inedible, parasitic fungus	HKAS-122599
*Hypomyces chrysospermus* Tul. & C. Tul	Hypocreaceae		**√**		Inedible, parasitic fungus	HKAS-122567
*Hypomyces perniciosus* Magnus	Hypocreaceae		**√**		Inedible, parasitic fungus	HKAS-111690
*Hypomyces pseudolactifluorum* F.M. Yu, Q. Zhao & K.D. Hyde	Hypocreaceae		**√**		Inedible, parasitic fungus	HKAS-122679
*Inocybe* sp.	Inocybaceae		**√**	**√**	Unknown	HKAS-123963
*Laccaria amethystina* Cooke	Hydnangiaceae		**√**	**√**	Edible	HKAS-122734
*Laccaria aurantia* Popa, Rexer, Donges, Zhu L. Yang & G. Kost	Hydnangiaceae		**√**	**√**	Edible	HKAS-122365
*Laccaria laccata* (Scop.) Cooke	Hydnangiaceae	**√**	**√**	**√**	Edible	HKAS-111743
*Laccaria moshuijun* Popa & Zhu Liang Yang	Hydnangiaceae		**√**	**√**	Edible	HKAS-122719
*Laccaria vinaceoavellanea* Hongo	Hydnangiaceae	**√**	**√**	**√**	Edible	HKAS-111721
*Laccaria yunnanensis* Popa, Rexer, Donges, Zhu L. Yang & G. Kost	Hydnangiaceae	**√**	**√**	**√**	Edible	HKAS-123996
*Lactarius acerrimus* Britzelm	Russulaceae		**√**	**√**	Edible, but not tasty	HKAS-111712
*Lactarius aff. subplinthogalus*	Russulaceae	**√**	**√**	**√**	Edible	HKAS-111825
*Lactarius akahatsu* Nobuj. Tanaka	Russulaceae	**√**	**√**	**√**	Edible	HKAS-122497
*Lactarius austrotorminosus* H.T. Le & Verbeken	Russulaceae		**√**	**√**	Edible	HKAS-122639
*Lactarius cinnamomeus* W.F. Chiu	Russulaceae		**√**	**√**	Edible	HKAS-122463
*Lactarius conglutinatus* X.H. Wang	Russulaceae		**√**	**√**	Toxic	HKAS-111697
*Lactarius formosus* H.T. Le & Verbeken	Russulaceae		**√**	**√**	Unknown	HKAS-111772
*Lactarius glabrigracilis* Wisitr. & Nuytinck	Russulaceae		**√**	**√**	Unknown	HKAS-111699
*Lactarius gracilis* Hongo	Russulaceae		**√**	**√**	Unknown	HKAS-111829
*Lactarius hatsudake* Nobuj. Tanaka	Russulaceae	**√**	**√**	**√**	Edible	HKAS-111725
*Lactarius hirtipes* J.Z. Ying	Russulaceae		**√**	**√**	Toxic	HKAS-122708
*Lactarius purpureus* R. Heim	Russulaceae		**√**	**√**	Edible, but not tasty	HKAS-111745
*Lactarius rubrobrunneus* H.T. Le & Nuytinck	Russulaceae	**√**	**√**	**√**	Edible	HKAS-111805
*Lactarius* sp.	Russulaceae		**√**	**√**	Edible	HKAS-122654
*Lactifluus* aff. *tropicosinicus*	Russulaceae		**√**	**√**	Edible	HKAS-122728
*Lactifluus ambicystidiatus* X.H. Wang	Russulaceae		**√**	**√**	Maybe inedible, bitter and spicy	HKAS-122435
*Lactifluus dwaliensis* (K. Das, J.R. Sharma & Verbeken) K. Das	Russulaceae	**√**		**√**	Edible	HKAS-111781
*Lactifluus gerardii* (Peck) Kuntze	Russulaceae	**√**	**√**	**√**	Edible	HKAS-122402
*Lactifluus hygrophoroides* (Berk. & M.A. Curtis) Kuntze	Russulaceae	**√**	**√**	**√**	Edible	HKAS-123965
*Lactifluus leae* (D. Stubbe & Verbeken) Verbeken	Russulaceae		**√**	**√**	Edible	HKAS-111695
*Lactifluus pilosus* (Verbeken, H.T. Le & Lumyong) Verbeken	Russulaceae	**√**	**√**	**√**	Edible	HKAS-111859
*Lactifluus pinguis* (Van de Putte & Verbeken) Van de Putte	Russulaceae	**√**	**√**	**√**	Edible	HKAS-122422
*Lactarius piperatus* (L.) Pers	Russulaceae	**√**	**√**	**√**	Edible	HKAS-111795
*Lactifluus pseudoluteopus* (X.H. Wang & Verbeken) X.H. Wang	Russulaceae		**√**	**√**	Maybe toxic	HKAS-122349
*Lactifluus rugatus* (Kühner & Romagn.) Verbeken	Russulaceae	**√**	**√**	**√**	Edible	HKAS-111848
*Lactifluus subpruinosus* X.H. Wang	Russulaceae		**√**	**√**	Edible	HKAS-122371
*Lactifluus volemus* (Fr.) Kuntze	Russulaceae	**√**	**√**	**√**	Edible	HKAS-122387
*Lanmaoa pallidorosea* (Both) Raspé & Vadthanarat	Boletaceae	**√**		**√**	Edible	HKAS-123971
*Lauriomyces heliocephalus* (V. Rao & de Hoog) R.F. Castañeda & W.B. Kendr	Lauriomycetaceae		**√**		Inedible, pathogenic fungus	HKAS-111894
*Leccinellum quercophilum* M. Kuo	Boletaceae		**√**	**√**	Edible	HKAS-122418
*Leccinum rugosiceps* (Peck) Singer	Boletaceae		**√**	**√**	Edible	HKAS-122386
*Lentinula edodes* (Berk.) Pegler	Omphalotaceae	**√**			Edible	HKAS-111768
*Lentinus squarrosulus* Mont	Omphalotaceae	**√**	**√**		Edible	HKAS-111758
*Leotia atrovirens* Pers	Leotiaceae		**√**		Unknown	HKAS-111847
*Leotia lubrica* (Scop.) Pers	Leotiaceae		**√**		Edible, but tasteless	HKAS-111791
*Lyophyllum fumosum* (Pers.) P.D. Orton	Lyophyllaceae	**√**		**√**	Edible	HKAS-111813
*Lyophyllum rhopalopodium* Clémençon	Lyophyllaceae		**√**	**√**	Unknown	HKAS-111793
*Macowanites chlorinosmus* A.H. Sm. & Trappe	Russulaceae		**√**		Unknown	HKAS-122489
*Macrocybe gigantea* (Massee) Pegler & Lodge	Callistosporiaceae	**√**			Edible	HKAS-122496
*Macrolepiota velosa* Vellinga & Zhu L. Yang	Agaricaceae		**√**		Unknown	HKAS-122634
*Marasmius* sp.	Marasmiaceae		**√**		Unknown	HKAS-111705
*Marasmius pseudopurpureostriatus* Wannathes, Desjardin & Lumyong	Marasmiaceae	**√**	**√**		Edible, but not worthwhile because of small size and thin flesh	HKAS-123994
*Microporus xanthopus* (Fr.) Kuntze	Polyporaceae		**√**		Inedible, leathery flesh	HKAS-111716
*Micropsalliota furfuracea* R.L. Zhao, Desjardin, Soytong & K.D. Hyde	Agaricaceae		**√**		Toxic	HKAS-122485
*Micropsalliota globocystis* Heinem	Agaricaceae		**√**		Unknown	HKAS-111724
*Nigroporus vinosus* (Berk.) Murrill	Steccherinaceae		**√**		Inedible, wood-decay fungus	HKAS-111839
*Neoboletus multipunctatus* N.K. Zeng, H. Chai & S. Jiang	Boletaceae		**√**	**√**	Unknown	HKAS-111883
*Ophiocordyceps nutans* (Pat.) G.H. Sung, J.M. Sung, Hywel-Jones & Spatafora	Ophiocordycipitaceae	**√**	**√**		Medicinal	HKAS-122621
*Ophiocordyceps oxycephala* (Penz. & Sacc.) G.H. Sung, J.M. Sung, Hywel-Jones & Spatafora	Ophiocordycipitaceae	**√**	**√**		Medicinal	HKAS-123960
*Panellus pusillus* (Pers. ex Lév.) Burds. & O.K. Mill	Mycenaceae		**√**		Inedible, maybe medicinal	HKAS-122667
*Panus tigrinus* (Bull.) Singer	Polyporaceae	**√**			Edible	HKAS-123984
*Paxillus involutus* (Batsch) Fr	Paxillaceae		**√**	**√**	Toxic	HKAS-122442
*Phaeocollybia pseudofestiva* A.H. Sm	Hymenogastraceae		**√**	**√**	Unknown	HKAS-111858
*Phaeocollybia ratticauda* E. Horak	Hymenogastraceae		**√**	**√**	Unknown	HKAS-111769
*Phaeocollybia redheadii* Norvell	Hymenogastraceae		**√**	**√**	Unknown	HKAS-111780
*Phaeolus schweinitzii* (Fr.) Pat	Fomitopsidaceae		**√**		Inedible, too tough	HKAS-122400
*Pholiota multicingulata* E. Horak	Strophariaceae		**√**	**√**	Maybe toxic	HKAS-122568
*Phylloporus luxiensis* M. Zang	Boletaceae		**√**	**√**	Edible	HKAS-111881
*Phylloporus rubiginosus* M.A. Neves & Halling	Boletaceae		**√**	**√**	Unknown	HKAS-122582
*Pisolithus tinctorius* (Mont.) E. Fisch	Sclerodermataceae		**√**	**√**	Medicinal	HKAS-123964
*Pluteus septocystidiatus* Ševčíková, Antonín & Borov	Pluteaceae		**√**		Unknown	HKAS-111864
*Podoscypha involuta (*Klotzsch ex Fr.) Imazeki	Podoscyphaceae		**√**		Unknown	HKAS-111782
*Polyporus cuticulatus* Y.C. Dai, Jing Si & Schigel	Polyporaceae	**√**	**√**		Edible	HKAS-111809
*Pulveroboletus icterinus* (Pat. & C.F. Baker) Watling	Boletaceae		**√**	**√**	Toxic, maybe medicinal	HKAS-111741
*Pulveroboletus subrufus* N.K. Zeng & Zhu L. Yang	Boletaceae		**√**	**√**	Toxic	HKAS-122514
*Ramaria asiatica* (R.H. Petersen & M. Zang) R.H. Petersen	Gomphaceae		**√**	**√**	Edible	HKAS-123983
*Ramaria cartilaginea* Marr & D.E. Stuntz	Gomphaceae	**√**	**√**	**√**	Edible	HKAS-123998
*Ramaria cyanocephala* (Berk. & M.A. Curtis) Corner	Gomphaceae		**√**	**√**	Maybe toxic	HKAS-122630
*Ramaria fennica* (P. Karst.) Ricken	Gomphaceae		**√**	**√**	Edible, but bitter	HKAS-111790
*Ramaria flava* (Schaeff.) Quél	Gomphaceae		**√**	√	Edible, but little bitter	HKAS-111706
*Ramaria pallida* (Schaeff.) Ricken	Gomphaceae	**√**	**√**	**√**	Edible	HKAS-123982
*Ramaria sanguinipes* R.H. Petersen & M. Zang	Gomphaceae		**√**	**√**	Edible	HKAS-111746
*Ramaria* sp.	Gomphaceae	**√**		**√**	Edible	HKAS-111774
*Ramaria thindii* K. Das, Hembrom, A. Parihar & A. Ghosh	Gomphaceae		**√**	**√**	Edible	HKAS-122425
*Ramaria vinosimaculans* Marr & D.E. Stuntz	Gomphaceae		**√**	**√**	Edible	HKAS-111785
*Retiboletus fuscus* (Hongo) N.K. Zeng & Zhu L. Yang	Boletaceae		**√**	**√**	Edible	HKAS-122545
*Retiboletus sinensis* N.K. Zeng & Zhu L. Yang	Boletaceae		**√**	**√**	Edible	HKAS-122610
*Retiboletus* sp.	Boletaceae		**√**	**√**	Unknown	HKAS-122552
*Rhizocybe alba* Y.X. Ding & E.J. Tian	Agaricales		**√**		Maybe toxic	HKAS-122720
*Rhizopogon songmaodan* R. Wang & Fu Q. Yu	Rhizopogonaceae	**√**		**√**	Edible	HKAS-123980
*Rubroboletus esculentus* Kuan Zhao, H.M. Shao & Zhu L. Yang	Boletaceae	**√**		**√**	Edible	HKAS-124003
*Rugiboletus extremiorientalis* (Lj.N. Vassiljeva) G. Wu & Zhu L. Yang	Boletaceae	**√**		**√**	Edible	HKAS-123978
*Russula adusta* (Pers.) Fr	Russulaceae		**√**	**√**	Edible	HKAS-122583
*Russula amarissima* Romagn. & E.-J. Gilbert	Russulaceae	**√**		**√**	Edible	HKAS-111737
*Russula cerea* (Soehner) J.M. Vida	Russulaceae		**√**	**√**	Unknown	HKAS-122509
*Russula compacta* Frost	Russulaceae	**√**	**√**	**√**	Edible	HKAS-111734
*Russula crustosa* Peck	Russulaceae		**√**	**√**	Edible	HKAS-122506
*Russula cyanoxantha* (Schaeff.) Fr	Russulaceae	**√**	**√**	**√**	Edible	HKAS-122577
*Russula delica* Fr	Russulaceae	**√**	**√**	**√**	Edible	HKAS-123987
*Russula densifolia* Secr. ex Gillet	Russulaceae		**√**	**√**	Edible	HKAS-122430
*Russula dissimulans* Shaffer	Russulaceae		**√**	**√**	Edible	HKAS-122628
*Russula flavida* Frost ex Peck	Russulaceae		**√**	**√**	Edible	HKAS-122512
*Russula foetens* Pers	Russulaceae		**√**	**√**	Toxic	HKAS-111702
*Russula griseocarnosa* X.H. Wang, Zhu L. Yang & Knudsen	Russulaceae	**√**	**√**	**√**	Edible	HKAS-122424
*Russula lakhanpalii* A. Ghosh, K. Das & R.P. Bhatt	Russulaceae		**√**	**√**	Unknown	HKAS-122622
*Russula lilacea Quél*	Russulaceae	**√**	**√**	**√**	Edible	HKAS-111853
*Russula nigricans* Fr	Russulaceae	**√**	**√**	**√**	Edible	HKAS-123961
*Russula purpureogracilis* F. Hampe, Looney & Manz	Russulaceae		**√**	**√**	Unknown	HKAS-111722
*Russula rosea* Pers	Russulaceae	**√**	**√**	**√**	Edible, but some consider it inedible	HKAS-122342
*Russula senecis* S. Imai	Russulaceae	**√**	**√**	**√**	Edible	HKAS-122352
*Russula* sp.	Russulaceae		**√**	**√**	Unknown	HKAS-122376
*Russula sororia* (Fr.) Romell	Russulaceae		**√**	**√**	Edible	HKAS-122487
*Russula substriata* J. Wang, X.H. Wang, Buyck & T. Bau	Russulaceae		**√**	**√**	Unknown	HKAS-122625
*Russula virescens* (Schaeff.) Fr	Russulaceae	**√**	**√**	**√**	Edible	HKAS-122384
*Russula viridicinnamomea* F. Yuan & Y. Song	Russulaceae		**√**	**√**	Edible	HKAS-122524
*Russula vinosa* Lindblad	Russulaceae	**√**	**√**	**√**	Edible	HKAS-122380
*Sarcoporia polyspora* P. Karst	Sarcoporiaceae		**√**		Inedible, woody-decay fungus	HKAS-122725
*Schizophyllum commune* Fr	Schizophyllaceae	**√**			Edible and medicinal	HKAS-123962
*Scleroderma flavidum Ellis & Everh*	Sclerodermataceae		**√**	**√**	Toxic	HKAS-122469
*Scleroderma sinnamariense* Mont	Sclerodermataceae		**√**	**√**	Toxic	HKAS-111718
*Scleroderma yunnanense* Y. Wang	Sclerodermataceae	**√**	**√**	**√**	Edible	HKAS-111786
*Scleroderma* sp.	Sclerodermataceae		**√**	**√**	Unknown	HKAS-111776
*Sparassis* sp.	Sparassidaceae		**√**		Unknown	HKAS-122536
*Stereopsis radicans* (Berk.) D.A. Reid	Stereopsidaceae		**√**	**√**	Unknown	HKAS-111876
*Strobilomyces confusus* Singer	Boletaceae		**√**	**√**	Edible	HKAS-122534
*Strobilomyces latirimosus* J.Z. Ying	Boletaceae		**√**	**√**	Edible	HKAS-122520
*Strobilomyces seminudus* Hongo	Boletaceae		**√**	**√**	Edible	HKAS-111720
*Stropharia rugosoannulata* Farl. ex Muriil	Strophariaceae		**√**		Edible	HKAS-122474
*Sulzbacheromyces yunnanensis* D. Liu, Li S. Wang & Goffinet	Lepidostromataceae		**√**		Unknown	HKAS-122355
*Suillellus* sp.	Boletaceae		**√**	**√**	Unknown	HKAS-111890
*Suillellus subvelutipes* (Peck) Murrill	Boletaceae		**√**	**√**	Maybe toxic	HKAS-111754
*Suillus bovinus* (L.) Roussel	Suillaceae		**√**	**√**	Edible	HKAS-111891
*Suillus luteus* (L.) Roussel	Suillaceae		**√**	**√**	Toxic	HKAS-111788
*Suillus placidus* (Bonord.) Singer	Suillaceae		**√**	**√**	Toxic	HKAS-122590
*Tapinella panuoides* (Fr.) E.-J. Gilbert	Tapinellaceae		**√**		Toxic	HKAS-122726
*Termiticola* sp.	Agaricaceae		**√**		Unknown	HKAS-111738
*Termitomyces albiceps* S.C. He	Lyophyllaceae	**√**	**√**		Edible	HKAS-111703
*Termitomyces aurantiacus* (R.Heim) R. Heim	Lyophyllaceae	**√**	**√**		Edible	HKAS-122633
*Termitomyces clypeatus* R. Heim	Lyophyllaceae	**√**	**√**		Edible	HKAS-123988
*Termitomyces eurrhizus* (Berk.) R. Heim	Lyophyllaceae	**√**	**√**		Edible	HKAS-124007
*Termitomyces heimii* Natarajan	Lyophyllaceae	**√**			Edible	HKAS-123975
*Termitomyces fuliginosus* R. Heim	Lyophyllaceae	**√**	**√**		Edible	HKAS-111732
*Termitomyces microcarpus* (Berk. & Broome) R. Heim	Lyophyllaceae	**√**	**√**		Edible	HKAS-111735
*Termitomyces* sp.1	Lyophyllaceae	**√**			Edible	HKAS-122510
*Termitomyces* sp.2	Lyophyllaceae	**√**			Edible	HKAS-122623
*Termitomyces striatus* (Beeli) R. Heim	Lyophyllaceae	**√**	**√**		Edible	HKAS-124012
*Thelephora ganbajun* M. Zang	Thelephoraceae	**√**	**√**	**√**	Edible	HKAS-111698
*Thelephora regularis* Schwein	Thelephoraceae	**√**	**√**	**√**	Edible	HKAS-111874
*Thelephora sikkimensis* K. Das, Hembrom & Kuhar	Thelephoraceae		**√**	**√**	Unknown	HKAS-122715
*Thelephora* sp.	Thelephoraceae	**√**	**√**	**√**	Edible	HKAS-111830
*Thelephora vialis* Schwein	Thelephoraceae	**√**	**√**	**√**	Edible	HKAS-122373
*Trichaptum abietinum* (Pers. ex J.F. Gmel.) Ryvarden	Hymenochaetales		**√**		Inedible, leathery flesh	HKAS-122706
*Tricholoma albobrunneum* (Pers.) P. Kumm	Tricholomataceae		**√**	**√**	Toxic	HKAS-122501
*Tricholoma equestre* (L.) P. Kumm	Tricholomataceae	**√**	**√**	**√**	Toxic, but sold in market	HKAS-111762
*Tricholoma fulvocastaneum* Hongo	Tricholomataceae	**√**		**√**	Edible	HKAS-106954
*Tricholoma olivaceum* Reschke, Popa, Zhu L. Yang & G. Kost	Tricholomataceae		**√**	**√**	Unknown	HKAS-122580
*Tricholoma saponaceum* (Fr.) P. Kumm	Tricholomataceae	**√**	**√**	**√**	Mild toxic, but sold in market	HKAS-111763
*Trogia infundibuliformis* Berk. & Broome	Marasmiaceae		**√**		Edible	HKAS-122453
*Turbinellus floccosus* (Schwein.) Earle ex Giachini & Castellano	Gomphaceae	**√**	**√**	**√**	Edible	HKAS-122519
*Tylopilus balloui* (Peck) Singer	Boletaceae		**√**	**√**	Toxic	HKAS-122578
*Tylopilus neofelleus* Hongo	Boletaceae	**√**	**√**	**√**	Toxic, but sold in market	HKAS-123985
*Tylopilus vinosobrunneus* Hongo	Boletaceae		**√**	**√**	Toxic	HKAS-111693
*Xylaria brevipes* Sacc. & Fairm	Xylariaceae		**√**		Medicinal	HKAS-122468

**Fig. 3:**
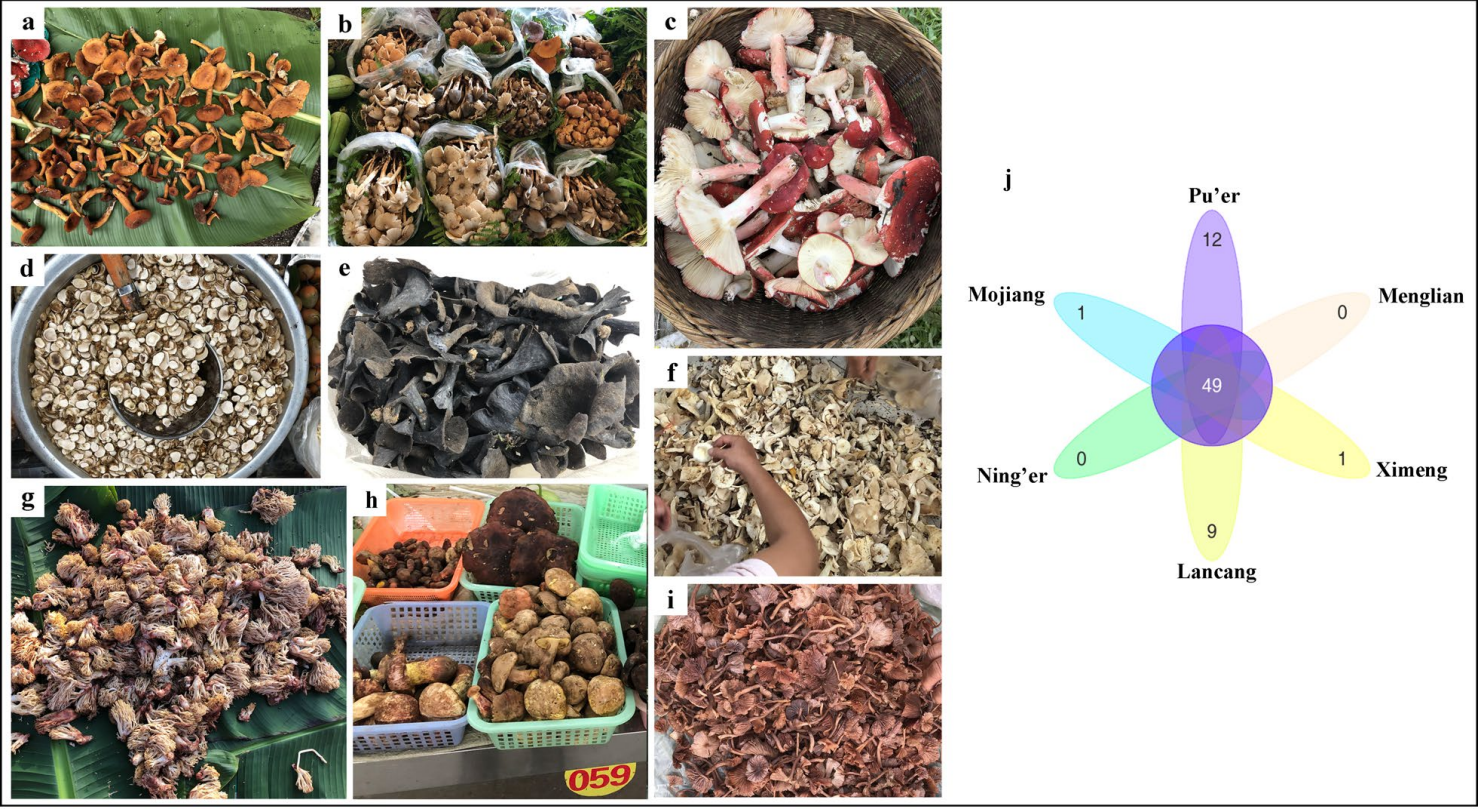
Popular species sold in the studied markets. **a** *Lactifluus volemus*; **b** *Termitomyces* spp.; **c** *Russula griseocarnosa*; **d** *Scleroderma yunnanense*; **e** *Craterellus cornucopioides*; **f** *Lactifluus piperatus*; **g** *Ramaria* spp.; **h** *Boletus* spp.; **i** *Laccaria laccata*; **j** Flower plot diagram showing shared and unique edible wild mushroom species in the sampled markets

In the markets, 91 species are edible and about 80% are EMF. A few new species which have only been published in recent years [44–47] were found in markets. And some previously described species were revised or classified in other section or genus by molecular phylogenetic study [48–50]. Furthermore, four species from markets are medicinal, two of which, *Ophiocordyceps nutans* (Pat.) G.H. Sung, J.M. Sung, Hywel-Jones & Spatafora and *O. oxycephala*, are mainly distributed in tropical and subtropical broad-leaved forests. It is interesting that four species which have been reported to cause gastroenteritis type poisoning, including *Heimioporus japonicus* (Hongo) E. Horak and *Tylopilus neofelleus* Hongo (in July) were sold in large quantities in Pu’er market, and *Tricholoma equestre* (L.) P. Kumm. (August to October) was mixed with a few *Tricholoma saponaceum* (Fr.) P. Kumm. in some small stalls. Some specimens, of one inedible species, *Abortiporus biennis* (Bull.) Singer, were recorded to be sold in a few markets as *Thelephora ganbajun* M. Zang*.* Similarly, *Hygrocybe cuspidata* (Peck) Murrill with unknown toxicity was sold occasionally in some stalls maybe because for some people it is *Cantharellus*-looking. Therefore, the accurate taxonomic status of these apparently toxic species has to be carefully checked, in order to determine if they correspond to new taxa or if the ecotypes in the area are non-toxic species. Most commercial mushrooms are common species in all markets (Fig. 3a–i). Six sampled markets shared 49 mushroom species, while 12 unique species were only sold in Pu’er market and 9 unique species were only sold in Lancang market (Fig. 3j).

**Fig. 4:**
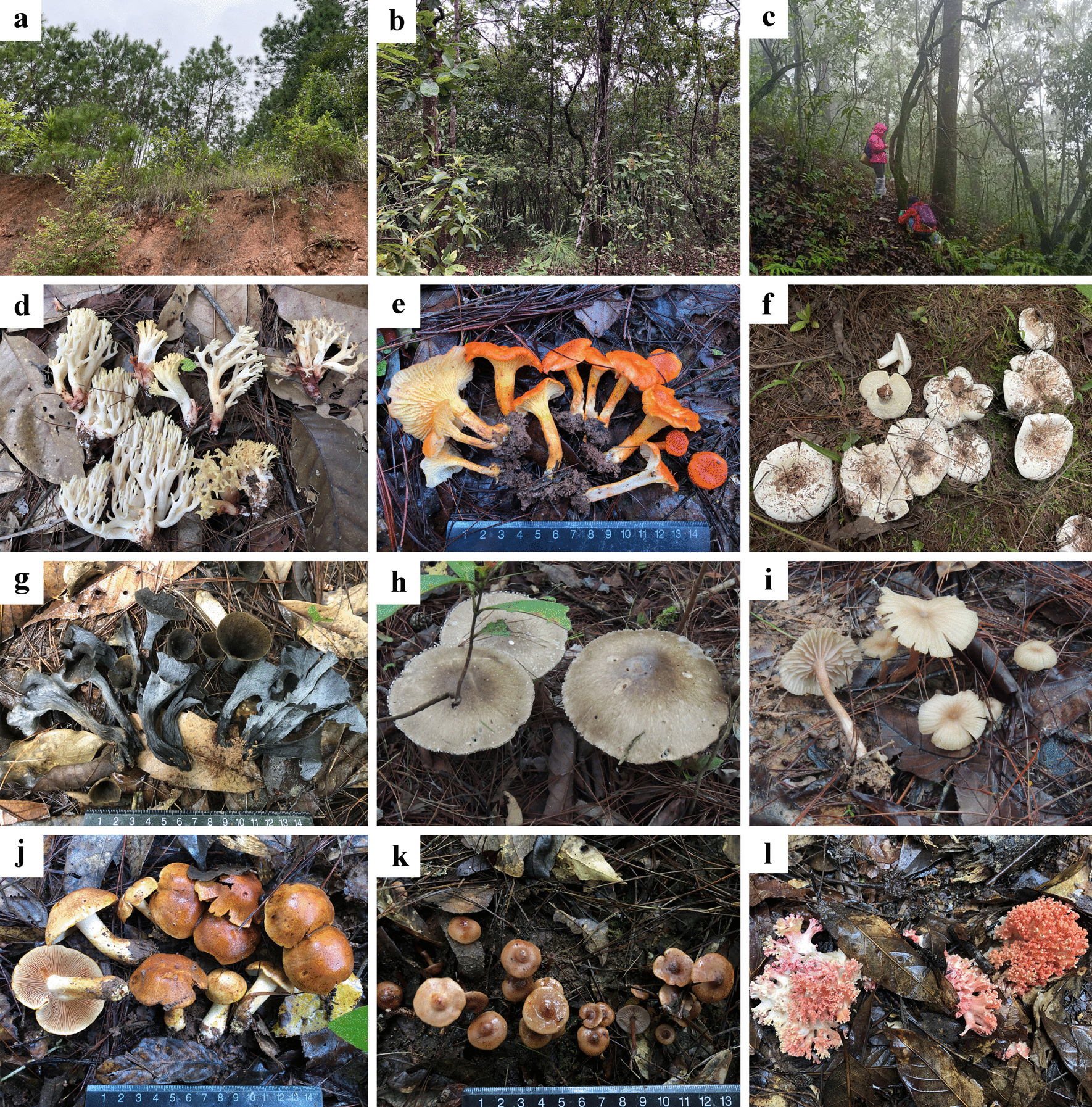
Typical edible wild mushrooms and their natural habitats. **a**–**c** Sampled vegetation types: **a** *Pinus kesiya* forest; **b** Coniferous and broad-leaved forest mixed forest; **c** Broad-leaved forest. **d**–**i** Representative abundant mushroom species: **d** *Ramaria* sp.; **e** *Cantharellus cinnabarinus*; **f** *Lactifluus piperatus*; **g** *Craterellus cornucopioides*; **h** *Amanita caojizong*; **i** *Laccaria yunnanensis*. **j**–**l** Some undescribed fungi: **j** *Cortinarius* sp.; **k** *Phaeocollybia* sp.; **l** *Ramaria* sp

The forest areas selected for the natural habitats work (according to information gathered from some collectors) were within 15 km of the markets. Due to its protected status, the Ecological Conservation Forests and the Sun River National Forest Park are less visited by gatherers or recreational visitors. A total of 283 species were recorded and collected from natural habitats, which include 129 edible species, accounting for about 84% EMF, 15 inedible species, 11 medicinal species, 53 poisonous species and 75 species with unknown edibility. Moreover, 23 species are undescribed and are currently under taxonomic study (Fig. 4).

#### Local preference and acceptability of WEF species

**Fig. 5:**
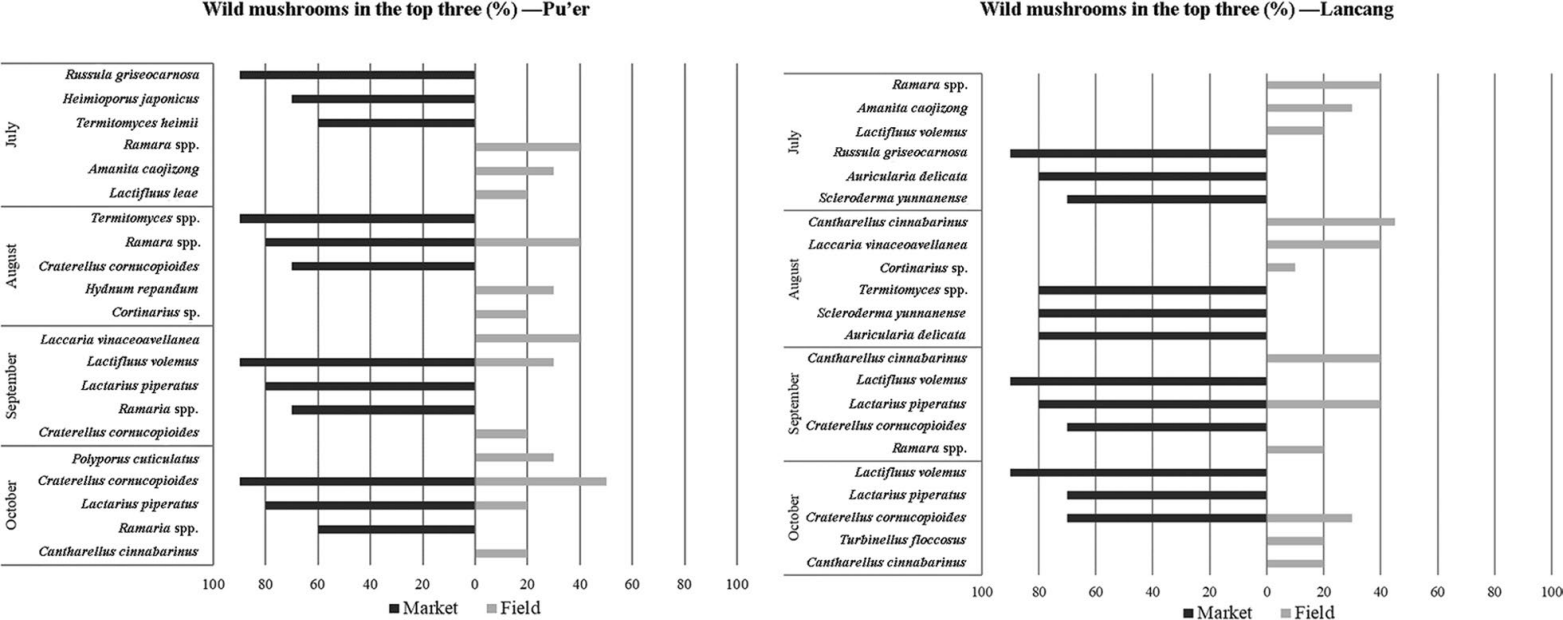
Comparison of the most abundant edible wild mushrooms sampled in markets (black bars) and natural habitats (gray bars) of Pu’er Municipality and Lancang County during July to October. Numbers of black bars represent percentages of stalls sold the most abundant edible wild mushrooms to total stalls; numbers of gray bars represent percentages of numbers of collected the most abundant edible wild mushrooms to total collected mushrooms

A total of 74 species were recorded in both markets and natural habitats, including 65 edible species, 4 medicinal species, 4 toxic species and one species with unknown use. *Amanita caojizong* Zhu L. Yang, Y.Y. Cui & Q. Cai, *Cantharellus cinnabarinus* (Schwein.) Schwein, *Craterellus cornucopioides* (L.) Pers., *Laccaria yunnanensis* Popa, Rexer, Donges, Zhu L. Yang & G. Kost, *Lactifluus piperatus* (L.) Pers., *Lactifluus volemus* (Fr.) Kuntze and *Ramaria* spp. were popular in markets and easy to find in natural habitats in mushroom season (Fig. 5). The most frequently bought wild mushrooms belonged mainly to Boletaceae (16 species), Hydnaceae (14 species), Lyophyllaceae (11 species) and Russulaceae (23 species). The families Amanitaceae (26 species), Boletaceae (32 species), Cortinariaceae (16 species), Hydnaceae (24 species), Hydnangiaceae (6 species), Lyophyllaceae (11 species) and Russulaceae (50 species) were common in natural habitats and forests. Mushroom species and amount showed a high correspondence between markets and the natural habitats on different months (Fig. 5). Preference of WEF for locals was mostly related to their availability in the forests.

### Ethnomycological data

#### Type of markets and constitution of vendors

A total of 11 markets were visited during this study. As illustrated in Table 2, three markets were established markets, 3 markets were mobile markets and 5 street-stalls were without names. Different markets have different sale time to sell mushrooms according to the local people’s different lifestyles. The highest number of vendors in all markets was recorded in August and September. The vendors in the mobile markets and in the street-stalls were usually low-income people, who travel usually by foot from the natural collection areas to the selling points.

Almost all vendors were able to speak Mandarin in Wuyi market of Pu’er City although most of vendors belong to ethnic groups, like Hani, Yi and Lahu people. This is the largest market in Pu’er, and up to 200 vendors, including gatherers, two-way merchants (those who buy mushrooms from gatherers directly in natural habitats) and brokers (those who buy mushrooms from gatherers or to two-way merchants), sold mushrooms in August and September (Fig. 2a–c). Most of the valuable mushrooms are usually sold at higher prices to large markets or restaurants of Kunming (the capital of Yunnan province) by brokers. Vendor’s main age group was between 35 and 55, and most of them were able to receive contactless payments through their mobile phones.

In the markets of Mojiang and Ning’er Counties, the number of vendors reached 100 in August or September. The Yi and Hani people are the main ethnic groups who inhabit these two counties. In recent years, local governments have paid great attention to the development of WEF resources marketing, and more mushroom markets have been established. Vendors in these markets were gatherers, merchants and some brokers, and the main age group was between 20 and 45. A small group of aged vendors (60+) spoke southwest Mandarin and could not use mobile phone apps to receive payments for the mushroom sale.

Lancang Street Market (Fig. 2d, e) had mostly Lahu and Hani people. The villagers in the surrounding towns bring a variety of products to Lancang Street Market on Sunday every week. Vendor’s main age group was between 40 and 65, 48% of which could not speak Mandarin, only Lahu language and southwest Mandarin. In addition, most of aged vendors accepted cash only. Nearly all vendors in Lancang Street Market were gatherers, and most of them usually sell mushrooms along with vegetables, fruits or local products, so seller mobility in this market was not strong within market time.

Menglian and Ximeng Counties are not far from Lancang County. The Lahu, Dai and Wa people are the main ethnic groups in these two counties. Vendors here spoke Lahu, Dai and Wa languages and southwest Mandarin. Compared with other markets, fewer vendors sold mushrooms. However, some vendors said that many buyers from Lancang County or Pu’er Municipality came here to buy mushrooms to process or dry and then resell them, so many vendors collect mushrooms and sell them directly to wholesale buyers.

Mushroom species from five street-stalls which have 1 to 15 vendors by county highways or village roads were also recorded. These vendors come from nearby villages and most of them were aged people. They do not have transportation to go to markets to sell mushrooms, so they usually sell them on the side of the road after collecting them. As a consequence, only very fresh mushrooms were recorded (Fig. 2 g, i).

#### Gender of vendors

The male-to-female ratio of vendors showed that women outnumber men in markets. Female vendors were involved in every stage of mushroom utilization from collection to processing and marketing.

#### Mushroom prices in three years

**Fig. 6:**
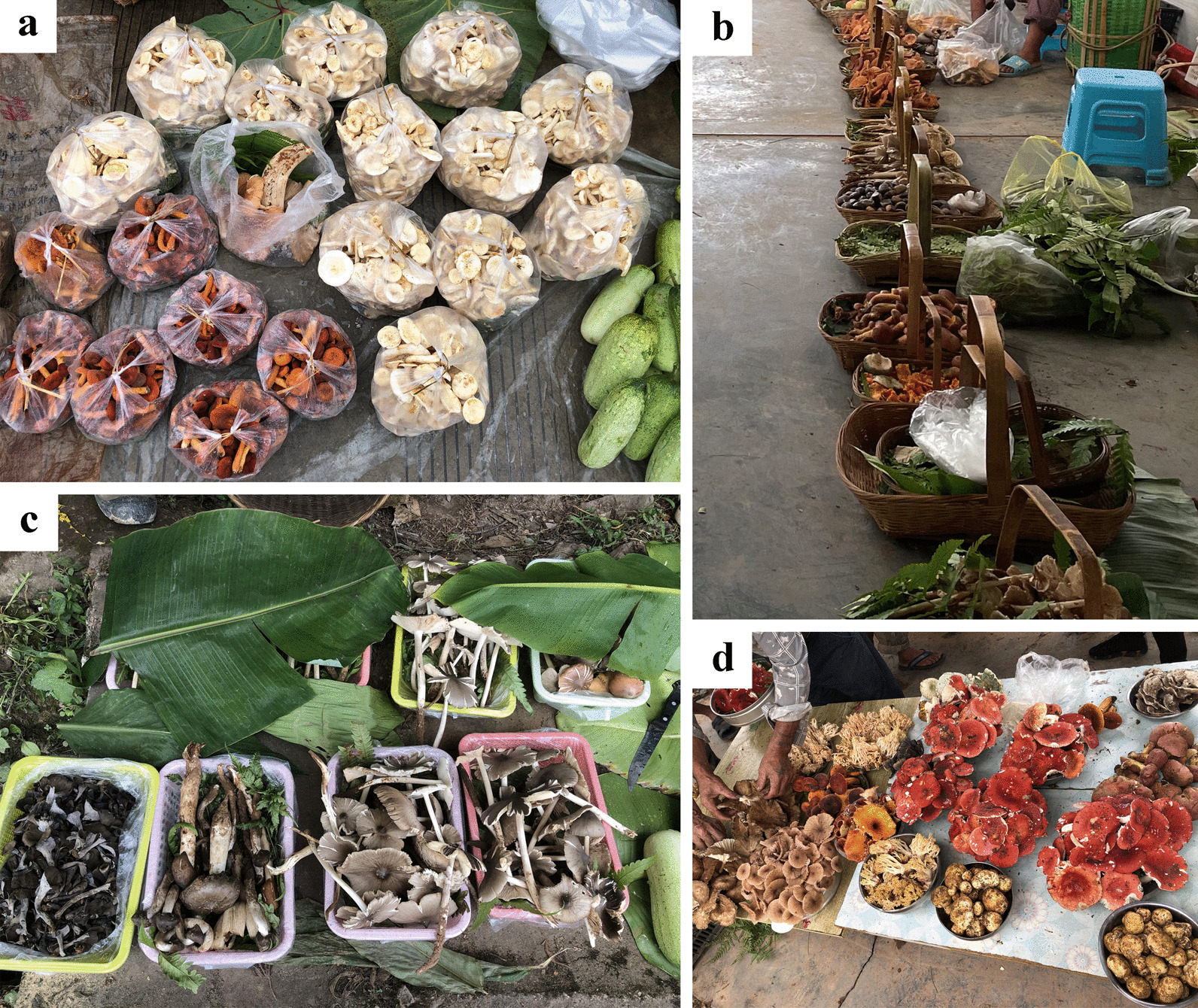
In each market, vendors place mushrooms on green banana leaves, plastic bags, baskets or cans with a defined weight (usually 0.5 kg or 1 kg), which facilitates the selling process

The prices of popular mushrooms were similar in the six studied counties, and the price of each species did not fluctuate much over the three years (Table 5). However, a large fluctuation was recorded throughout the mushroom season mainly due to their availability and quality of the specimens. Overall, the prices of popular mushrooms, *Russula griseocarnosa* X.H. Wang, Zhu L. Yang & Knudsen, *Termitomyces* spp. (e.g., *T. globulus*, *T. striatus*) and *Thelephora ganbajun* were higher than those of other mushroom species. *Schizophyllum commune* Fr. was only recorded to be sold in a few stalls in each market, and its price was as high as to 200 yuan per kilogram. In each market, vendors carefully placed mushrooms on green banana leaves or in plastic bags, baskets or plates (Fig. 6) with a certain weight (generally 0.5 kg or 1 kg), which due to the arrangement always looked beautiful and clean.

**Table 5:** Sale prices of frequently bought mushrooms as recorded in 2019 to 2021

Species	Year 2019	Year 2020	Year 2021
*Boletus* spp. (porcini group)	30–90 yuan/kg	20–75 yuan/kg	30–85 yuan/kg
*Craterellus cornucopioides*	20–70 yuan/kg	20–60 yuan/kg	20–70 yuan/kg
*Laccaria* spp.	20–40 yuan/kg	15–40 yuan/kg	15–40 yuan/kg
*Lactifluus piperatus*	10–30 yuan/kg	10–30 yuan/kg	10–30 yuan/kg
*Lactifluus volemus*	30–90 yuan/kg	25–90 yuan/kg	30–90 yuan/kg
*Ramaria* spp.	20–40 yuan/kg	20–40 yuan/kg	10–40 yuan/kg
*Russula griseocarnosa*	45–120 yuan/kg	50–120 yuan/kg	40–130 yuan/kg
*Russula virescens*	30–80 yuan/kg	30–90 yuan/kg	30–80 yuan/kg
*Scleroderma yunnanense*	15–40 yuan/kg	15–40 yuan/kg	15–40 yuan/kg
*Termitomyces* spp.	30–160 yuan/kg	30–160 yuan/kg	25–160 yuan/kg
*Thelephora ganbajun*	90–180 yuan/kg	95–180 yuan/kg	90–195 yuan/kg

Except for brokers, most collectors are farmers who grow tea and other crops or raise hogs and cattle. During the mushroom season, they usually collect wild mushrooms in the mountains near their homes and sell them for an extra income (3000–6000 yuan per family, approximately equivalent to USD$450 to 900) for their families.

#### The use and preparation methods of WEF

**Fig. 7:**
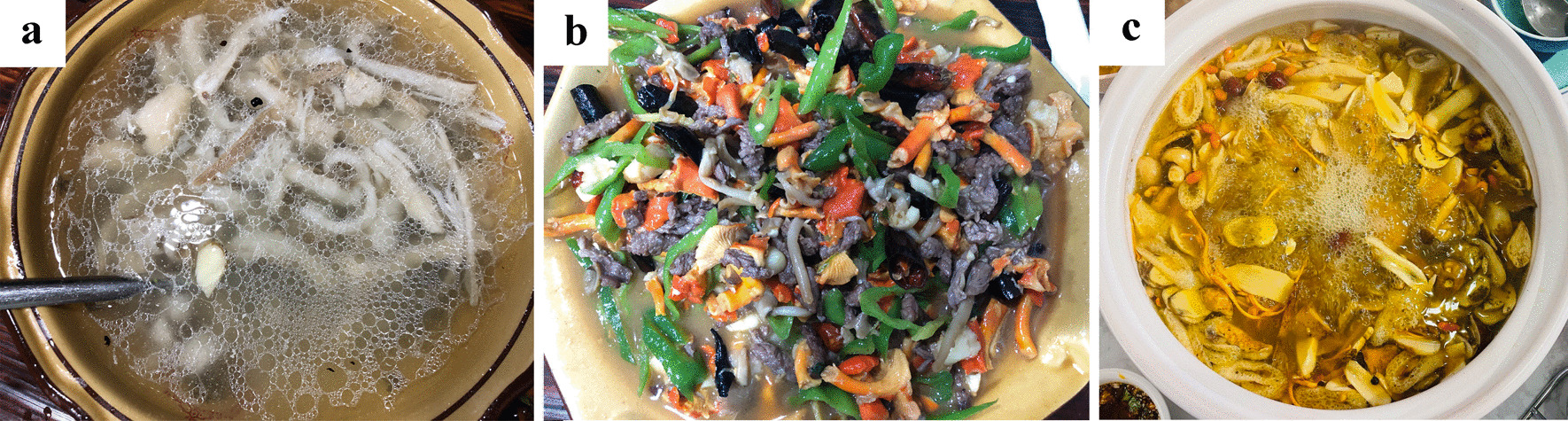
Preparation way of wild fungi. **a** *Termitomyces* soup; **b** Stir-fried *Cantharellus cinnabarinus*; **c** Hot pot with Boletaceae, *Lactarius*, *Lyophyllum*, *Russula* and some artificial cultivated mushrooms

The main use of wild mushrooms is for food, and a few are medicinal species used to make medicinal liquor (Fig. 7, Table 6). The most common cooking preparation way among local people was to fry the mushrooms with fermented bamboo shoots or other local vegetables. *Lactifluus piperatus,* which has a spicy taste, is considered to be a perfect match for sour pickles. *Tylopilus neofelleus* is an interesting species considered toxic by some local people; however, other people enjoy its bitter flavor. They found a cooking method to remove toxic components, which was by drying slices of the mushroom and then deep frying them. For species of Boletaceae, local people had a common understanding of adding more garlic and cooking them for more than 30 min. Likewise, local people soak peeled *Scleroderma yunnanense* Y. Wang fruiting bodies or slices in water or saline water before cooking to remove some components to avoid any gastrointestinal upset.

**Table 6:** Local preferred preparations and storage methods for edible mushrooms

Species	Preparation	Note	Storage
*Amanita caojizong*, *A. sinensis*	Make soup, stir-fry with little garlic	−	−
Boletaceae	Fried with garlic and chili (dry chili or fresh chili)	Cooking time must be longer	Slice and dry Fry and soak in oil
*Cantharellus* spp.	Stir-fried with little garlic	−	Dry
*Craterellus cornucopioides*	Stir-fry with garlic and chili	Cooking time is short to keep its crisp mouthfeel	−
*Lactifluus piperatus*	Chop mushrooms, then fry with garlic, dry chili and sour bamboo shoots or pickles	−	−
*Lactifluus volemus*	Fry with garlic, chili and meat	−	−
*Ramaria* spp.	Fry with garlic, dry chili and sour bamboo shoots or pickles	−	Dry
*Russula griseocarnosa*	Cook with chicken soup	−	Dry
*Russula virescens*	Stir-fry with garlic and fresh chili Cook with meat soup	−	Dry
*Scleroderma yunnanense*	Slice, fry with garlic and chili	Peel and soak in water before cooking to reduce bitter taste	Slice and pickle with salt
*Termitomyces* spp.	Make soup Fried mushroom oil	−	Fry and soak in oil
*Thelephora ganbajun*	Fried with garlic, chili and bacon	−	−
*Tylopilus neofelleus*	Dry, slice and deep fry	−	−

Local people stored mushrooms by drying, pickling and frying, but they enjoy more to eat fresh mushrooms. Some dry mushrooms, like *Boletus* spp. (porcini), *Russula griseocarnosa*, *Russula virescens* (Schaeff.) Fr. and *Ramaria* spp., were usually sold to people from other cities.

#### Traditional recognition methods of WEF

The rich variety of mushroom species gathered by local people demonstrate that they have a rich traditional knowledge. Local mushroom names demonstrate a particular taxonomic knowledge. According to the color, shape, taste, texture, habitat and some special features of mushrooms or even local legends, interesting and vivid names have been given to mushrooms and people are able to make a local classification system for mushrooms (Table 7). Sometimes, mushrooms have more than one name, like *Scleroderma yunnanense*, which is named “bubble with horse skin” in most areas of Pu’er because of its shape and texture, but Lahu people call it “soil fruits” because of its habitat. *Lactifluus rugatus* (Schaeff.) Fr. is named “milk cap mushroom” because of the fluid it produces, and the names “monkey mushroom” (local monkeys are yellow) and “sweet yellow mushroom” come from its pileus color and taste. Experienced gatherers have a more impressive knowledge. Such as valuable *Russula griseocarnosa* could be distinguished from other similar or poisonous species by its thick pileus, light-gray context and solid stipe (they usually squeeze the stipe). *Amanita caojizong* and poisonous *Amanita pseudoporphyria* Hongo are locally distinguished by the stipe shape and smell. The knowledge of selecting mushrooms has usually passed from generation to generation. Moreover, some collectors have their own mental maps to find specific places where mushrooms, especially valuable ones, appear every year, and the information is usually kept within their family to avoid the collection by other people, which would affect their family’s income.

**Table 7:**
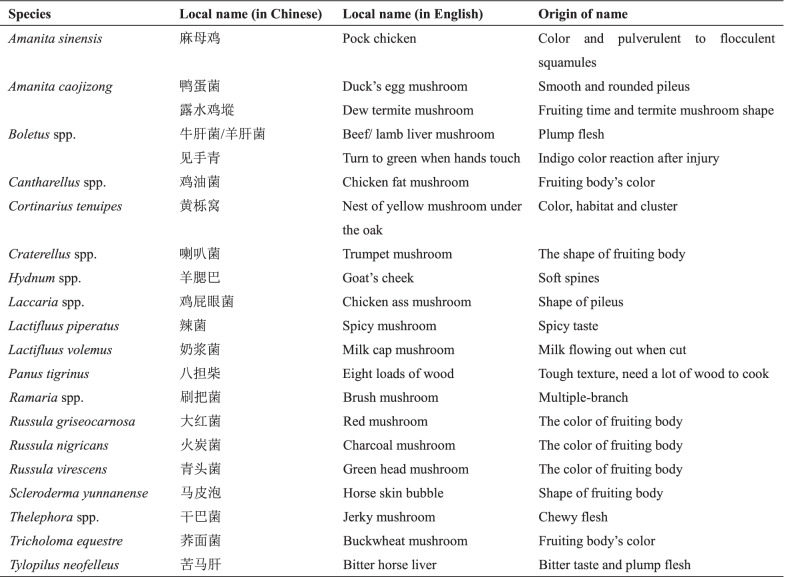
Interesting local name of popular commercial mushrooms in markets

## Discussion

A total of 310 wild mushroom species, varieties and some undescribed species were collected from markets and natural areas. Approximately 70% of the species were ectomycorrhizal. In the markets from the 91 edible species, about 80% were EMF. With the development of transportation infrastructures, Pu’er has become one of the main supply centers of WEF for central Yunnan, and WEF processing industries are becoming large scale. Yu et al. [33] surveyed markets in Pu’er from 2002 to 2009 and reported a sharp decline of WEF production of 43 species, such as *Lactifluus volemus*, *Russula griseocarnosa*, *Termitomyces* spp. and *Thelephora ganbajun* which were considered important in Yunnan. In our study, interviews with vendors showed that production of these species had declined even more in recent years and they had to travel farther to collect them. However, we also found that some mushrooms, that were not so common then, are now popular in Pu’er area, such as *Cantharellus cinnabarinus*, *Laccaria laccata* and *Boletus edulis* [33]. These species have a high market value and high production in the sites sampled in our study. This change might have due to the growing mycological knowledge of Pu’er people. The increase in mushroom species could reduce the pressure of collection of valuable species to some extent. But local people still act cautiously and even refuse eating some edible mushrooms that have only recently become mainstream edibles. In our study, 57 good edible species that we found in nature were not sold in markets. Very tasty species as *Amanita subhemibapha* Zhu L. Yang, Y.Y. Cui & Q. Cai, *Boletus violaceofuscus* W.F. Chiu and *Laccaria amethystina* Cooke have beautiful color and good production in the forests, but they were not recorded in the markets maybe due to the fact that they are preferred for self-consumption rather than commercialization. The utilization of WEF resources in Pu’er still has great potential to be developed. However, in the studied area the knowledge and implementation of strategies and actions in order to protect the decline of relevant WEF are its infancy. In general, fungi have historically been left out of conservation initiatives [51]. In addition, climate change, habitat loss, overexploitation and land pollution might be affecting the natural production of WEF. Therefore, it is urgent for the development of ecological studies and the implementation of comprehensive monitoring of natural production of WEF in the studied area along with cultivation of ectomycorrhizal edible fungi. These strategies would allow the development of codes of conduct and appropriate legislation related to the maximum amounts allowed to be collected for marketing, optimal harvesting methods and sustainable use of the relevant genetic resource constituted by WEF in the area.

In our study, some poisonous mushrooms were identified. Mushroom poisoning has always been an important food safety issue in China, and it recently has gained a conspicuous attention. Currently, the Chinese Centers for Disease Control and Prevention have developed a systematic technical support network supported by technical staff, doctors and mycologists. This has allowed to start a precise record of the mushroom species involved in mushroom poisoning in the country. Li et al. [52–54] identified using morphological and molecular characterization, approximately 74 poisonous mushrooms which originated hundreds mushroom incidents in 25 provinces up to now. The most dangerous mushrooms, *Amanita exitialis*, *Lepiota brunneoincarnata* and *Russula subnigricans,* showed the highest fatality rate. Seven different mycetism syndromes have been recorded worldwide [45]: acute liver failure, acute renal failure, rhabdomyolysis, gastroenteritis, psychoneurological disorder, erythrolysis and photosensitive dermatitis, all of which have been recorded in China [55, 56]. Despite the fact that very complete reviews have been published dealing with the mycetisms and their potential treatments [57, 58], the topic is far to be complete. With the advent of molecular techniques, new poisonous species continue being identified [59, 60]. Therefore, a more active role of scientists, doctors and policy-makers at local and national levels is urgently necessary in order to reduce mycetisms in China and worldwide.

A total of 11 markets from one municipality and 5 counties were visited during this study. Sales activities of wild mushrooms can be carried out uniformly in established markets, while local government strengthens the sales supervision of markets to make the sale of wild mushrooms more standardized and reduce the probability of wild mushroom poisoning. In each market, the male-to-female ratio of vendors showed that women outnumber men. It seems that in many regions of the world women are often the main collectors [61–63]. But women usually collected mushrooms closest to their houses, while men go farther to collect. For this reason, men usually have developed a deeper knowledge related to WEF compared to women. We recorded that the members of the local ethnic groups have developed a profound knowledge in order to distinguish edible species from those poisonous ones. This knowledge is based on accurate morphological characterization, ecological observations, association with vegetation types or even specific trees and phenological patterns of WEF. In addition, the age of collectors was mainly between 45 and 65 years old and only few young people were involved in mushroom collecting or selling. Traditional knowledge is being lost through economic change, modernization, urbanization and even formal education. Therefore, further research on ethnomycology is urgent to preserve the current knowledge before their lost forever.

Despite the fact that open-air markets in southeast Asia are relevant reservoirs of biocultural diversity in southeast Asia, they have been largely understudied. As far as useful mycological resources concerns, it has been shown that these markets are additionally an important source of traditional knowledge due to the fact that frequently the sellers are the current gatherers, recipients of ancestral mycological knowledge. Some areas in different parts of southwest Asia have shown to harbor a great biodiversity of edible mushrooms. For example, in the markets of Luang Prabang in north Central Laos, 54 species of fungi have been reported to be sold [64]. In this area, a large number of rare species of *Russula,* some probably new to science, are commercialized in local markets. Some of the species reported from markets of this region of Laos were also recorded to be sold in the Pu’er’s studied markets in our work including: *Amanita princeps*, *Auricularia delicata*, *Boletus reticulatus*, *Lactifluus pinguis*, *Lentinula edodes*, *Lentinus squarrosulus*, *Macrocybe gigantea*, *Russula delica*, *R. virescens*, *Schizophyllum commune*, *Termitomyces fuliginosus*, *T. eurhizus*, *T. heimii* and *T. microcarpus* [64]. Recently, a monograph of the useful fungi of Northern Laos, including edible and medicinal species has been published [65]. There are also a large number of species reported in this monograph with those sold in the markets of Pu’er in China. These include members of the genera *Amanita, Auricularia, Boletus Cantharellus, Craterellus, Lactarius, Lactifluus, Lentinus, Lentinula, Lyophyllum, Ramaria, Russula, Thelephora, Termitomyces, Tricholoma* and *Tylopilus*, most of which are EMF. The situation of the ethnomycological understudy of open-air markets selling wild mushrooms is not exclusive of Southeast Asia, but it is a global issue. For example in Tanzania, 128 edible wild mushrooms are commercialized in 31 traditional markets. Among them the genera with the highest diversity were *Termitomyces, Cantharellus* and *Russula* with 21, 17 and 9 species, respectively [66]. In Mexico, in one single market located in the central part of the country, called Ozumba, 60 species of WEF were reported to be sold. In this market, with 411 stands selling WEF mainly during July and August, 90% of the vendors were women, and 64% were between 40 and 60 years old [62]. In southeastern Poland, 30 edible wild mushrooms were recorded to be sold [67]. A similar number of species have been recorded to be sold in western Black Sea region in Turkey, where 33 edible wild mushrooms are commercialized in 70 local markets [21]. In other areas, a smaller number of species have been recorded to be sold in open-air markets; for example, in Armenia located in Western Asia, only 12 edible wild species of mushrooms have been reported to be commercialized [68]. In fact, in general the open-air markets constitute cultural treasures, whose study should receive more attention in order to increase the knowledge related to the sustainable use and conservation of wild mushrooms as a paramount local source of food around the globe.

## Conclusion

We recorded a wealth of ethnomycological knowledge through interviews and collected abundant wild mushroom samples from local markets and forests in three consecutive years. Mushroom harvesting is a challenging activity that requires a deep local environmental knowledge to achieve success. Local mushroom collectors in Pu’er have rich experience with the habitats where their WEF proliferate, their fruiting time and species identification which comes mainly from the previous generation, as well as special cooking and preservation methods. There are established markets, mobile markets and street-stalls for selling mushrooms in Pu’er area. In markets, men usually develop a more profound knowledge on WEF than woman, although the number of female vendors is larger than that of male vendors. Our current study provides useful documentation, which contributes to preserving ethnomycological knowledge in Pu’er Prefecture. In addition, the diversity of species of wild fungi, especially ectomycorrhizal fungi, in markets and natural areas has been updated and supplemented which helps us to recognize mushroom species accurately and detect valuable species. Local preference and acceptance of more mushroom species of WEF may reduce the pressure to collect traditional choice species. However, the rational management of WEF species with high yield in natural areas and the collection and use of ectomycorrhizal fungi germplasm resources for cultivation will benefit the sustainable utilization of local WEF. Finally, it is necessary to continue the research of ethnomycology in order to preserve existing knowledge, since knowledge of fungi remains mainly among the elderly population.

## Data Availability

Not applicable.
